# Pd-Catalyzed Amination in the Synthesis of a New Family of Macropolycyclic Compounds Comprising Diazacrown Ether Moieties

**DOI:** 10.3390/molecules19010940

**Published:** 2014-01-15

**Authors:** Alexei A. Yakushev, Nataliya M. Chernichenko, Maxim V. Anokhin, Alexei D. Averin, Alexei K. Buryak, Franck Denat, Irina P. Beletskaya

**Affiliations:** 1 Department of Chemistry, Lomonosov Moscow State University, Leninskie Gory, 1–3, Moscow 119991, Russia; E-Mails: longhauler@yandex.ru (A.A.Y.); natashachernichenko@mail.ru (N.M.C.); anokhinmv@gmail.com (M.V.A.); beletska@org.chem.msu.ru (I.P.B.); 2 A.N. Frumkin Institute of Physical Chemistry and Electrochemistry, 31 Leninskii prosp., Moscow 119991, Russia; E-Mail: akburyak@mail.ru; 3 Institut de Chimie Moléculaire de l’Université de Bourgogne (ICMUB), UMR CNRS 6302, 9 avenue A. Savary, Dijon Cedex 21078, France; E-Mail: fdenat@u-bourgogne.fr

**Keywords:** diazacrown ethers, polyamines, Pd catalysis, amination, macropolycycles

## Abstract

*N*,*N'*-bis(bromobenzyl) and *N*,*N'*-bis(halopyridinyl) derivatives of diaza-12-crown-4, diaza-15-crown-5 and diaza-18-crown-6 ethers were synthesized in high yields. The Pd-catalyzed macrocyclization reactions of these compounds were carried out using a variety of polyamines and oxadiamines were carried out to give novel macrobicyclic and macrotricyclic compounds of the cryptand type. The dependence of the yields of macropolycycles on the nature of the starting diazacrown derivatives and polyamines was established. Generally *N,N'*-bis(3-bromobenzyl)-substituted diazacrown ethers and oxadiamines provided better yields of the target products. The highest yield of the macrobicyclic products reached 57%.

## 1. Introduction

Macropolycyclic compounds (cryptands) attract the continued interest of researchers due to their unique selective ion binding properties. Macrobicycles of the cryptand type derived from azacrown ethers were among the first reported molecules of this type, e.g., di- and triazapolyoxacryptands [1,2], benzocryptands possessing 1,2-, 1,3-, and 1,4-disubstituted benzene [3,4], and 2,6-disubstituted pyridine fragments [5]. Compounds with two diazacrown ethers combined in macrotricyclic systems via aliphatic or benzyl linkers were also described [6,7]. So-called cross-bridged polycyclic compounds comprising diazacrown ethers constitute another class of cryptands called supercryptands [8]. Krakowiak and coauthors elaborated convenient and versatile synthetic approaches to various macropolycycles in 1990s based on simple nucleophilic substitution reactions [9,10,11]. Our interest in this field arises from the possibilities of the application of the catalytic Buchwald-Hartwig amination in the construction of the polymacrocyclic systems capable of selective metal cations coordination. We have already successfully used this approach for the synthesis of macrobicycles comprising tetraazamacrocyclic [12,13,14] moieties and made the first steps in the formation of polymacrocyclic structures based on aza- and diazacrown ethers [15,16].

## 2. Results and Discussion

**Scheme 1:**
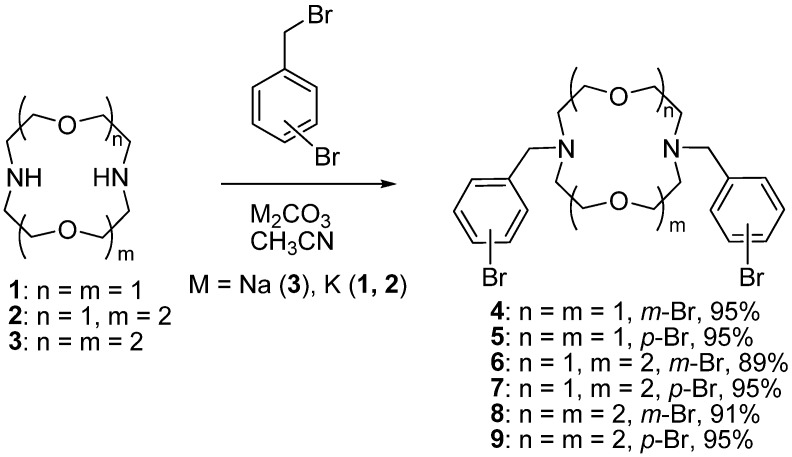
Synthesis of *N,N'*-bis(bromobenzyl) derivatives of diazacrown ethers **4**–**9**.

Initially we attempted to synthesize a series of macrobicycles possessing diaza-12-crown-4 moieties because these compounds are of interest for selective coordination of Li ions. The search for efficient macrocyclic chelators of this ion is important for the sequestration of ^7^Li and ^6^Li isotopes. It is well known that a partial change of oxygen for nitrogen atoms in 12-member macrocycles and introduction of podands to these nitrogen atoms increases the stability constants of the lithium complexes by 2–3 orders of magnitude [17,18], thus we might expect that the macrobicycles with additional donor atoms will also form more stable complexes with Li cations. At the first step we synthesized *N,N'*-bis(bromobenzyl) derivatives of diaza-12-crown-4 by reacting 1 equiv. of compound **1** with 2 equiv. of 3- and 4-bromobenzyl bromides in boiling acetonitrile using K_2_CO_3_ as a base. As a result, the corresponding derivatives **4** and **5** were obtained in almost quantitative yields (Scheme 1). The same method was applied for the modification of diaza-15-crown-5 (**2**) and diaza-18-crown-6 (**3**), and corresponding *N,N'*-bis(bromobenzyl) derivatives **6**–**9** were obtained in 89%–95% yields (Scheme 1). Na_2_CO_3_ was used as a base in the case of diaza-18-crown-6.

**Figure 1:**
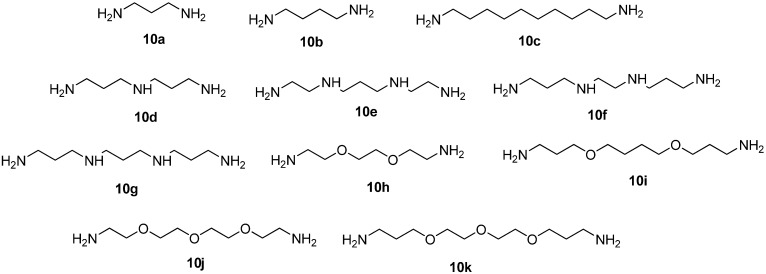
Polyamines and oxadiamines **10a**–**k** used in the synthesis of macrobicycles.

The macrocyclization reactions of compounds **4**–**9** were carried out using a series of di-, tri-, tetraamines, and oxadiamines **10a**–**k** differing in the chain length and the number of N and O atoms (Figure 1). The investigation of the extended set of polyamines was necessary for elucidation of the scope and limitations of the proposed method and for the construction of macrobicycles with various macrocyclic cavities what would be useful for tuning their coordination properties towards different metal cations.

**Scheme 2:**
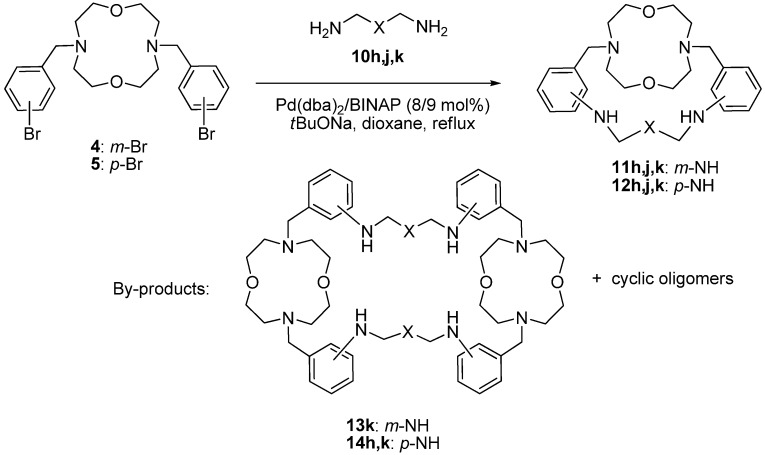
Synthesis of macrobicycles **11** and **12**.

Macrocycles **4** and **5** were introduced in the Pd-catalyzed amination reactions with oxadiamines **10h**,**j**,**k** using 8 mol% Pd(dba)_2_/BINAP catalytic system (dba—dibenzylideneacetone, BINAP = 2,2'-bis(diphenylphosphino)-1,1'-binaphthalene) which previously was shown to be optimal in the majority of the amination reactions of aryl halides, and especially in the macrocyclization processes involving polyamines. The syntheses were carried out in boiling dioxane (c = 0.02 M) using sodium *tert*-butoxide as a base (Scheme 2). Macrobicycles **11** and **12** were isolated by column chromatography on silica gel. The yields are given in Table 1.

We did not observe any correlation between the structures of the starting compounds and the yields of the macrobicycles, which ranged from 13% to 31%. In some cases macrotricyclic cyclodimers **13** and **14** were isolated, in yields comparable to those of the target compounds (Table 1, entries 3, 4, 6). In two cases mixtures of cyclic oligomers were obtained in yields *ca* 40% (entries 1, 2). These facts imply that in some cases the intramolecular diamination is hindered, probably due to unfavorable mutual orientation of two bromine atoms.

**Scheme 3:**
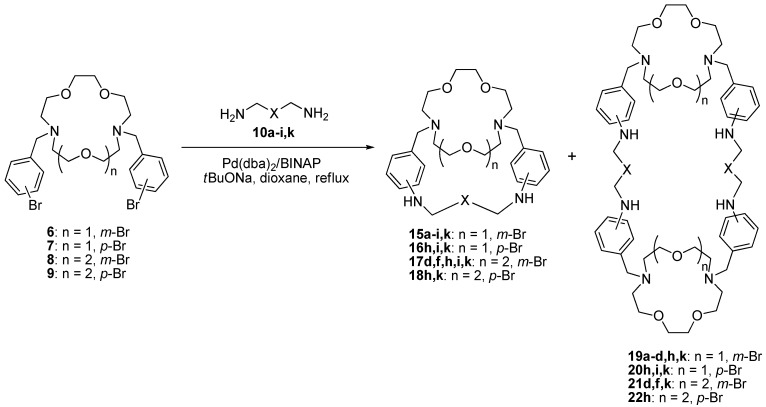
Synthesis of macrobicycles **15**–**18**.

Further investigations were carried out using *N,N'*-bis(3-bromobenzyl) derivative of diaza-15-crown-5 **6** and a wide range of polyamines to study the process in details and to find out scope and limitations of the proposed approach. The reactions were conducted under the same conditions using 8 mol% catalyst (Scheme 3), the results are presented in Table 2.

**Table 1:** Synthesis of macrobicycles **11** and **12**.

Entry	Diazacrown derivative	Polyamine	Yields of macrobicycles	Yields of cyclic oligomers
1	**4**	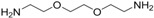 **10h**	**11h**, 13%	mixture, 42%
2	**4**	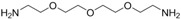 **10j**	**11j**, 31%	mixture, 37%
3	**4**	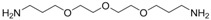 **10k**	**11k**, 19%	**13k**, 23%
4	**5**	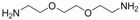 **10h**	**12h**, 30%	**14e**, 27%
5	**5**	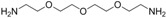 **10j**	**12j**, 20%	
6	**5**	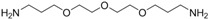 **10k**	**12k**, 15%	**14k**, 17%

In the majority of cases we obtained rather good yields of the target macrobicycles **15** ranging from 20% to 38%. The reactions with the shortest propane-1,3-diamine (**10a**) and butane-1,4-diamine (**10b**) gave poorer results (Table 2, entries 1, 2) due to the higher steric demands of these diamines for the mutual orientation of two bromine atoms in the starting compound **6**. For the rest of di- and polyamines we did not observe any clear dependence of the product yields on the chain length and on the number of the nitrogen and oxygen atoms. In many cases we managed to isolate macrotricyclic by-products **19**, and in the reaction with **10b** the yield of **19b** was twice as much as of the corresponding macrobicycle **15b**. In all cases we also obtained complex mixtures of cyclic oligomers but their composition cannot be unambiguously established by NMR and mass spectroscopies because they possess almost the same structural fragments.

**Table 2:** Synthesis of macrobicycles **15**–**18**.

Entry	Diazacrown derivative	Polyamine	Pd(dba)_2_/L,mol% ^a^	Yields of macrobicycles	Yields of cyclodimers
1	**6**	** 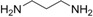 (10a)**	8/9	**15a**, 19%	**19a**, 19%
2	**6**	** 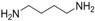 (10b)**	8/9	**15b**, 12%	**19b**, 21%
3	**6**	** 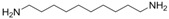 (10c)**	8/9	**15c**, 25%	**19c**, 15%
4	**6**	** 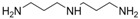 (10d)**	8/9	**15d**, 36%	**19d**, 9%
5	**6**	** 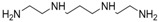 (10e)**	8/9	**15e**, 28%	
6	**6**	** 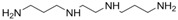 (10f)**	8/9	**15f**, 33%	
7	**6**	** 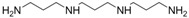 (10g)**	8/9	**15g**, 24%	
8	**6**	** 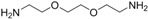 (10h)**	8/9	**15h**, 20%	**19h**, 10%
9	**6**	** 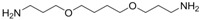 (10i)**	8/9	**15i**, 37%	
10	**6**	** 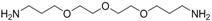 (10k)**	8/9	**15k**, 38%	**19k**, 24%
11	**7**	** 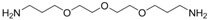 (10k)**	8/9	**16k**, 5%	**20k**, 10%
12	**7**	** 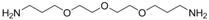 (10k)**	8/9	**16k**, 4%	**20k**, 10%
13	**7**	** 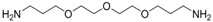 (10k)**	16/18	**16k**, 10%	**20k**, 19%
14	**7**	** 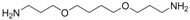 (10i)**	16/18	**16i**, 10%	**20i**, 10%
15	**7**	** 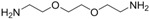 (10h)**	16/18	**16h**, 18%	**20h**, 31%
16	**8**	** 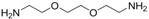 (10d)**	8/9	**17d**, 25%	**21d**, 6%
17	**8**	** 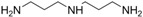 (10f)**	8/9	**17f**, 10%	**21f**, 5%
18	**8**	** 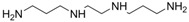 (10h)**	8/9	**17h**, 57%	
19	**8**	** 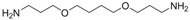 (10i)**	8/9	**17i**, 28%	
20	**8**	** 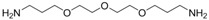 (10k)**	8/9	**17k**, 35%	**21k**, 17%
21	**9**	** 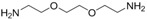 (10h)**	16/18	**18h**, 25%	**22h**, 10%
22	**9**	** 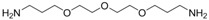 (10k)**	16/18	**18k**, 36%	

^a^ L = BINAP in all entries, except 12; in entry 12 L = DavePhos.

Other derivatives of diazacrown ethers **7**–**9** were tested mainly in the cyclization reactions with oxadiamines to establish the dependence of the product yields on the ring size and substitution patterns. The reactions of the isomeric diazacrown derivative **7** containing 4-bromobenzyl substituents provided substantially lower yields of the cryptands **16** (entries 11–15). Indeed, the use of the standard catalytic system in the macrocyclization reaction with trioxadiamine **10k** afforded only 5% yield of the desired macrobicycle **16k** (entry 11). The application of another ligand DavePhos (2-(dimethylamino)-2'-(dicyclohexylphosphino)biphenyl) was not successful either (entry 12), however, 16 mol% of the catalytic system Pd(dba)_2_/BINAP was helpful (entry 13) though the yield remained low. Only the reaction with dioxadiamine **10h** proceeded better and produced the cryptand **16h** in 18% yield (entry 15). In all cases the yields of cyclic dimers **20** exceeded those of macrobicycles **16**.

The macrocyclization reactions with the derivative of diaza-18-crown-6 **8** bearing two 3-bromobenzyl substituents were quite successful (entries 16–20) at 8 mol% catalyst loadings. While the use of triamine **10d**, oxadiamine **10i**,**k** provided average 25%–35% yields of the macrocyclization products **17d**,**i**,**k** (entries 16, 19, 20), the reaction with dioxadiamine **10h** resulted in 57% yield of the target cryptand **17h** (entry 18), what is the best result ever observed among yields in the Pd-catalyzed macrocyclization reactions. On the other hand, macrotricyclic dimers **21** were isolated in certain cases in much lower yields. The reactions with isomeric derivative **9** were run using a 16 mol% catalytic system (entries 21, 22) and the yields of the target cryptands **18** were quite reasonable. It means that of four tested *N*,*N'*-bis(bromobenzyl) substituted diazacrowns, only compound **7** was recalcitrant in the intramolecular diamination processes.

**Scheme 4:**
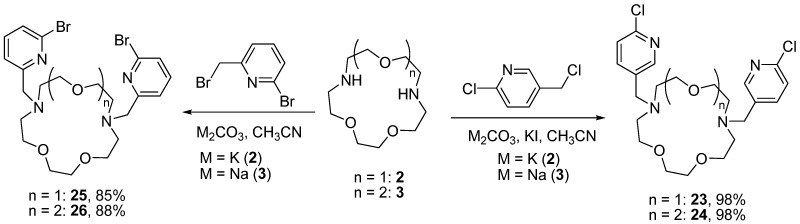
Synthesis of *N,N'*-bis(halopyridinyl) derivatives of diazacrown ethers **23**–**26**.

The incorporation of the pyridine moiety in the structure of macrocyclic compounds can be useful as it increases the number of donor sites of the molecule what is favorable for the complexation of the cations with high coordination numbers. We synthesized *N*,*N'*-bis(halopyridinyl) derivatives of diazacrown ethers **23**–**26** differing in the nature of the halogen atom and the position of the nitrogen atom (Scheme 4). The reactions were conducted in boiling acetonitrile using sodium or potassium carbonates as bases, and the yields of the target compounds were excellent.

**Scheme 5:**
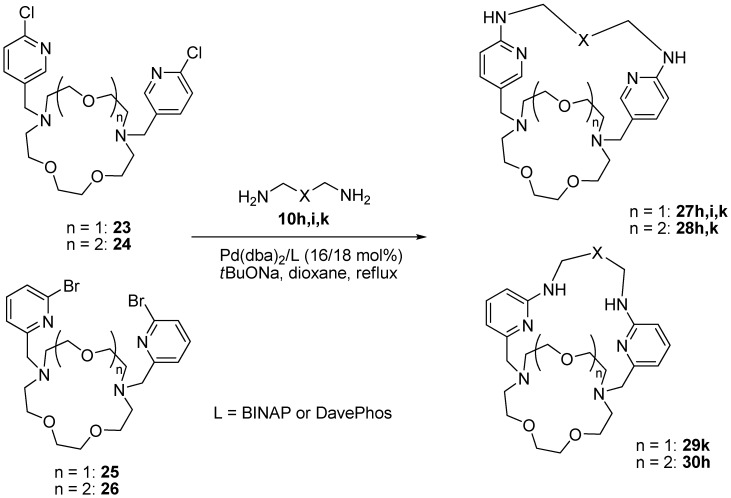
Synthesis of macrobicycles **27**–**30**.

All our attempts to induce the macrocyclization of compound **23** using the Pd(dba)_2_/BINAP catalytic system failed, however the application of DavePhos instead of BINAP was helpful (Scheme 5, Table 3). The same situation was observed with the derivative **24**, however, the yields of the target macrobicycles **27**, **28** were reasonable only in some cases (entries 1, 5). The analysis of the reaction mixtures and fractions after chromatography revealed the formation of complex mixtures of oligomers and other unidentified products which could arise from the side reactions other than catalytic amination. This is supported by the fact that the conversion of starting *N*,*N'*-bis(chloropyridinyl) derivatives **23** and **24** was complete whereas only half of the oxadiamines was consumed. Unfortunately, the efficiency of the bromosubstituted derivatives **25** and **26** to form macrobicycles was even poorer than that of compounds **23** and **24**. Only in the reactions of **25** with trioxadiamine **10k** and of **26** with dioxadiamine **10h** did yields exceed 10% (entries 6, 8), in other cases they were negligible and are not given in Table 3. A possible explanation is that bromine-containing derivatives **25** and **26** are more active than their chlorine-containing analogues **23** and **24** and participate in various side reactions. It is worth noting that both BINAP and DavePhos ligands can be used with limited success in the amination of compounds **25** and **26**.

**Table 3:** Synthesis of macrobicycles **27**–**30**.

Entry	Diazacrownderivative	Polyamine	Ligand L	Yields of macrobicycles
1	**23**	** 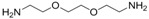 (10h)**	DavePhos	**27h**, 22%
2	**23**	** 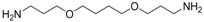 (10i)**	DavePhos	**27i**, 11%
3	**23**	** 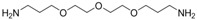 (10k)**	DavePhos	**27k**, 9%
4	**24**	** 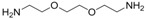 (10h)**	DavePhos	**28h**, 5%
5	**24**	** 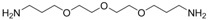 (10k)**	DavePhos	**28k**, 24%
6	**25**	** 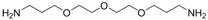 (10k)**	BINAP	**29k**, 12%
7	**26**	** 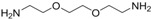 (10h)**	BINAP	**30h**, 9%
8	**26**	** 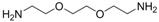 (10h)**	DavePhos	**30h**, 16%

To summarize, we have conducted an extended investigation of the scope of Pd-catalyzed amination in the synthesis of macrobicycles based on diazacrown ether moieties, and determined the dependence of the yields of the target cryptands on the nature of halogen-containing substituents in the starting compounds. The macrocyclization processes were shown to proceed more efficiently with *N*,*N'*-bis(3-bromobenzyl) substituted diazacrown ethers **6** and **8**, and the formation of valuable macrotricyclic compounds was demonstrated. The studies of the coordination properties of novel macrobicycles towards different metal cations are underway now.

## 3. Experimental

### 3.1. General Information

NMR spectra were registered using a Bruker Avance 400 spectrometer(operating at 400 MHz for ^1^H and 100.6 MHz for ^13^C), MALDI-TOF spectra were obtained with a Bruker Ultraflex spectrometer using 1,8,9-trihydroxyanthracene as matrix and PEGs as internal standards. ESI-TOF spectra were recorded with a Bruker microQ-TOF spectrometer in methanol. Diazacrown ethers, 3- and 4-bromobenzyl bromides, 2-chloro-5-(chloromethyl)pyridine, 2-bromo-6-methylpyridine, oxadiamines and polyamines, BINAP and DavePhos ligands, sodium *tert*-butoxide were purchased from Sigma-Aldrich (St. Louis, MO, USA) and used without further purification, Pd(dba)_2_ was synthesized from PdCl_2_ according to the known procedure [19]. 2-Bromo-6-(bromomethyl)pyridine was synthesized from 2-bromo-6-methylpyridine using a standard bromination procedure (Br_2_/CCl_4_/NBS). Dioxane was distilled over NaOH, followed by distillation over sodium under argon, while acetonitrile, dichloromethane and methanol were used freshly distilled.

### 3.2. General Method for the Synthesis of N,N'-bis(haloaryl)substituted Diazacrown Ethers

A one-neck flask equipped with a magnetic stirrer and reflux condenser was charged with diazacrown ether (0.86–2.3 mmol), aryl halide halogenomethyl derivative (1.7–4.6 mmol), dry acetonitrile (3–8 mL) and sodium or potassium carbonate (3.4–11.2 mmol). The reaction mixture was stirred under reflux for several hours, the residue was filtered off, washed with CH_2_Cl_2_, and the combined organic fractions were evaporated *in vacuo*, dissolved in CH_2_Cl_2_ (5–20 mL), washed three times with equal volumes of distilled water, dried over 4 Å molecular sieves, and the CH_2_Cl_2_ was evaporated *in vacuo* to give the pure target product. We have previously reported the synthesis and spectral data of compounds **6**–**9** [15].

*4,10-Bis(3-bromobenzyl)-1,7-dioxa-4,10-diazacyclododecane* (**4**). Obtained from diazacrown **1** (0.86 mmol, 150 mg), 3-bromobenzyl bromide (1.7 mmol, 431 mg) in the presence of K_2_CO_3_ (4.3 mmol, 530 mg) in MeCN (3 mL). Yield 419 mg (95%), as a yellowish viscous oil. ^1^H-NMR (CDCl_3_) δ 2.73 (d, ^3^*J* = 4.6 Hz, 8H, CH_2_N), 3.58 (t, ^3^*J* = 4.6 Hz, 8H, CH_2_O), 3.63 (s, 4H, PhCH_2_N), 7.17 (t, ^3^*J* = 7.8 Hz, 2H, H5(Ph)), 7.30–7.37 (m, 4H, H4(Ph), H6(Ph)), 7.58 (s, 2H, H2(Ph)). ^13^C-NMR (CDCl_3_) δ 55.0 (4C, CH_2_N), 60.3 (2C, PhCH_2_N), 69.4 (4C, CH_2_O), 122.3 (2C, C3(Ph)), 127.3 (2C, CH(Ph)), 129.7 (2C, CH(Ph)), 129.9 (2C, CH(Ph)), 131.7 (2C, CH(Ph)), 142.2 (2C, C1(Ph)). HRMS (MALDI-TOF): C_22_H_29_Br_2_N_2_O_2_ (M+H)^+^ calcd.; 511.0596 observed; 511.0632.

*4,10-Bis(4-bromobenzyl)-1,7-dioxa-4,10-diazacyclododecane* (**5**). Obtained from diazacrown **1** (0.86 mmol, 150 mg), 4-bromobenzyl bromide (1.7 mmol, 431 mg) in the presence of K_2_CO_3_ (4.3 mmol, 530 mg) in MeCN (3 mL). Yield 421 mg (95%), of a beige crystalline powder, m.p. 88–90 °C. ^1^H-NMR (CDCl_3_) δ 2.70 (t, *^3^J* = 4.7 Hz, 8H, CH_2_N), 3.56 (t, *^3^J* = 4.7 Hz, 8H, CH_2_O), 3.58 (s, 4H, PhCH_2_N), 7.28 (А part of АА'XX' system, 4H, H2(Ph)), 7.42 (X part of АА'XX' system, 4H, H3(Ph)). ^13^C-NMR (CDCl_3_) δ 54.9 (4C, CH_2_N), 60.2 (2C, PhCH_2_N), 69.4 (4C, CH_2_O), 120.5 (2C, C4(Ph)), 130.4 (4C, CH(Ph)), 131.2 (4C, CH(Ph)), 138.8 (2C, C1(Ph)). HRMS (MALDI-TOF): C_22_H_29_Br_2_N_2_O_2_ (M+H)^+^ calcd.; 511.0596 observed; 511.0557.

*7,13-Bis[(6-chloropyridin-3-yl)methyl]-1,4,10-trioxa-7,13-diazacyclopentadecane* (**23**). Obtained from diazacrown ether **2** (2.3 mmol, 500 mg), 2-chloro-5-(chloromethyl)pyridine (4.6 mmol, 745 mg) in the presence of K_2_CO_3_ (11.6 mmol, 1.6 g) in MeCN (8 mL). Yield 1.057 g (98%), of a yellow glassy compound. ^1^H-NMR (CDCl_3_) δ 2.71 (t, *^3^J* = 5.0 Hz, 4H, CH_2_N), 2.75 (t, *^3^J* = 5.8 Hz, 4H, CH_2_N), 3.52–3.57 (m, 8H, CH_2_O), 3.58 (s, 4H, CH_2_O or PyCH_2_N), 3.62 (s, 4H, PyCH_2_N or CH_2_O), 7.24 (d, *^3^J* = 8.1 Hz, 2H, H5-Py), 7.71 (dd, *^3^J* = 8.1 Hz, *^4^J* = 1.4 Hz, 2H, H6-Py), 8.29 (br.s, 2H, H2-Py). ^13^C-NMR (CDCl_3_) δ 54.3 (2C, CH_2_N), 54.4 (2C, CH_2_N), 57.0 (2C, PyCH_2_N), 69.3 (2C, CH_2_O), 70.1 (2C, CH_2_O), 70.6 (2C, CH_2_O), 123.9 (2C, C5-Py), 134.2 (2C, C1-Py), 139.4 (2C, C6-Py), 149.7 (2C, C2-Py), 150.0 (2C, C4-Py). HRMS (MALDI-TOF): C_22_H_31_Cl_2_N_4_O_3_ (M+H)^+^ calcd.; 469.1773 observed; 469.1742.

*1,16-Bis[(6-chloropyridin-3-yl)methyl]-1,4,10,13-tetraoxa-7,16-diazacyclooctadecane* (**24**). Obtained from diazacrown **3** (1 mmol, 262 mg), 2-chloro-5-(chloromethyl)pyridine (2 mmol, 324 mg) in the presence of K_2_CO_3_ (5 mmol, 690 mg) in MeCN (3 mL). Yield 504 mg (98%), of a yellow glassy compound. ^1^H-NMR (CDCl_3_) δ 2.75 (t, *^3^J* = 5.6 Hz, 8H, CH_2_N), 3.54 (s, 8H, CH_2_O), 3.56 (t, *^3^J* = 5.6 Hz, 8H, CH_2_O), 3.65 (s, 4H, PyCH_2_N), 7.22 (d, *^3^J* = 8.0 Hz, 2H, H5-Py), 7.67 (d, *^3^J* = 8.0 Hz, 2H, H6-Py), 8.27 (br.s, 2H, H2-Py). ^13^C-NMR (CDCl_3_) δ 53.7 (4C, CH_2_N), 56.2 (2C, CH_2_NPy), 69.7 (4C, CH_2_O), 70.6 (4C, CH_2_O), 123.8 (2C, C5-Py), 134.3 (2C, C1-Py), 139.3 (2C, C6-Py), 149.6 (2C, C2-Py), 149.8 (2C, C4-Py). HRMS (MALDI-TOF): C_24_H_35_Cl_2_N_4_O_4_ (M+H)^+^ calcd.; 513.2035 observed; 513.2052.

*4,13-Bis[(6-bromopyridin-2-yl)methyl]-1,7,10-trioxa-4,13-diazacyclohexadecane* (**25**). Obtained from diazacrown ether **2** (1 mmol, 218 mg), 2-bromo-6-(bromomethyl)pyridine (2 mmol, 502 mg) in the presence of K_2_CO_3_ (5 mmol, 690 mg) in MeCN (3 mL). Yield 474 mg (85%), of a yellow glassy compound. ^1^H-NMR (CDCl_3_) δ 2.67–2.77 (m, 8H, CH_2_N), 3.46-3.52 (m, 8H, CH_2_O), 3.53 (s, 4H, CH_2_O or PyCH_2_N), 3.72 (br.s, 4H, PyCH_2_N or CH_2_O), 7.24 (br.s, 2H, H-Py), 7.42 (br.s, 2H, H-Py), 7.53 (br.s, 2H, H-Py). ^13^C-NMR (CDCl_3_) δ 54.4 (2C, CH_2_N), 54.9 (2C, CH_2_N), 61.4 (2C, PyCH_2_N), 68.6 (4C, CH_2_O), 69.6 br (2C, CH_2_O), 122.3 br (2C, H6-Py), 126.3 (2C, H4-Py), 139.2 (2C, H5-Py), 141.2 (2C, C3-Py), 159.5 (2C, C1-Py). HRMS (MALDI-TOF): C_22_H_31_Br_2_N_4_O_3_ (M+H)^+^ calcd.; 557.0763 observed; 557.0722.

*7,16-Bis[(6-bromopyridin-2-yl)methyl]-1,4,10,13-tetraoxa-7,16-diazacyclooctadecane* (**26**). Obtained from diazacrown ether **3** (1 mmol, 262 mg) 2-bromo-6-(bromomethyl)pyridine (2 mmol, 502 mg) in the presence of K_2_CO_3_ (5 mmol, 690 mg) in MeCN (3 mL). Yield 530 mg (88%) of a yellow crystalline powder, m.p. 131–133 °С. ^1^H-NMR (CDCl_3_) δ 2.39 (br.s, 8H, CH_2_N), 3.29–3.33 (m, 8H, CH_2_O), 3.34 (br.s, 12H, CH_2_O, PyCH_2_N), 7.00 (d, *^3^J* = 7.8 Hz, 2H, H6-Py), 7.08 (d, *^3^J* = 7.8 Hz, 2H, H4-Py), 7.36 (t, *^3^J* = 7.7 Hz, 2H, H5-Py). ^13^C-NMR (CDCl_3_) δ 54.5 (4C, CH_2_N), 59.4 (2C, PyCH_2_N), 67.4 (4C, CH_2_O), 70.1 (4C, CH_2_O), 123.0 (2C, C6-Py), 126.4 (2C, C4-Py), 139.4 (2C, C5-Py), 141.3 (2C, C3-Py), 159.4 (2C, C1-Py). HRMS (MALDI-TOF): C_24_H_35_Br_2_N_4_O_4_ (M+H)^+^ calcd.; 601.1025 observed; 601.9973.

### 3.3. General Method for Palladium-Catalyzed Macrocyclizations

A two-neck flask equipped with a magnetic stirrer and reflux condenser, flushed with dry argon, was charged with diazacrown derivative **4**–**9** (0.2–0.25 mmol), Pd(dba)_2_ (8–16 mol%), BINAP or DavePhos ligand (9–18 mol%), absolute dioxane (10–12 mL), the reaction mixture was stirred for several minutes, then the corresponding polyamine (0.2–0.25 mmol) and NaO*t-*Bu (0.6–0.75 mmol) were added, and the reaction mixture was stirred at reflux for 24 h. After cooling down to room temperature the residue was filtered off, washed with CH_2_Cl_2_ (5–10 mL), the combined organic fractions were evaporated *in vacuo*, the residue was dissolved in CH_2_Cl_2_ (10 mL), washed with distilled water (3 × 10 mL), dried over 4Å molecular sieves, and the solvent was evaporated *in vacuo*. The solid residue was chromatographed on silica gel (40–60 μm) using a sequence of eluents: CH_2_Cl_2_, CH_2_Cl_2_–MeOH 100:1–3:1, CH_2_Cl_2_–MeOH–NH_3_(aq) 100:20:1–10:4:1.

*11,14,27,32-Tetraoxa-1,8,17,24-tetraazatetracyclo[22.5.5.1^3,7^.1^18,22^]hexatriaconta-3(36),4,6,18(35),19,21-hexaene* (**11h**). Obtained from compound **4** (0.2 mmol, 102 mg), dioxadiamine **10h** (0.2 mmol, 30 mg) in the presence of Pd(dba)_2_ (18 mg, 16 mol%), BINAP (22 mg, 18 mol%), NaO*t-*Bu (0.6 mmol, 57 mg) in abs. dioxane (10 mL). Eluent CH_2_Cl_2_–MeOH–NH_3_(aq) = 100:20:2. Yield 13 mg (13%), of a yellowish viscous oil. ^1^H-NMR (CDCl_3_) δ 2.76 (br.s, 8H, CH_2_N), 3.32 (t, *^3^J* = 4.9 Hz, 4H, CH_2_NPh), 3.58 (br.s, 12H, CH_2_O, PhCH_2_N), 3.66 (s, 4H, CH_2_O), 3.72 (t, *^3^J* = 4.9 Hz, 4H, CH_2_O), 4.17 (br.s, 2H, NH), 6.46–6.54 (m, 4H, H4(Ph), H6(Ph)), 6.98 (s, 2H, H2(Ph), 7.06 (t, *^3^J* = 7.7 Hz, 2H, H5(Ph)). ^13^C-NMR (CDCl_3_) δ 43.8 (2C, CH_2_NPh), 55.3 (4C, CH_2_N), 62.0 (2C, PhCH_2_N), 69.5 (4C, CH_2_O), 69.7 (2C, CH_2_O), 70.4 (2C, CH_2_O), 111.3 (2C, CH(Ph)), 114.5 (2C, CH(Ph)), 118.2 (2C, CH(Ph)), 128.8 (2C, C5(Ph)), 139.8 (2C, C1(Ph)), 148.7 (2C, C3(Ph)). HRMS (MALDI-TOF): C_28_H_43_N_4_O_4_ (M+H)^+^ calcd.; 499.3284 observed; 499.3251.

*11,14,17,30,35-Pentaoxa-1,8,20,27-tetraazatetracyclo[25.5.5.1^3,7^.1^21,25^]nonatriaconta-3(39),4,6,21 (38),22,24-hexaene* (**11j**). Obtained from compound **4** (0.2 mmol, 102 mg), trioxadiamine **10j** (0.2 mmol, 38 mg) in the presence of Pd(dba)_2_ (9 mg, 8 mol%), BINAP (11 mg, 9 mol%), NaO*t-*Bu (0.6 mmol, 57 mg) in abs. dioxane (10 mL). Eluent CH_2_Cl_2_–MeOH–NH_3_(aq) = 100:20:1. Yield 34 mg (31%), yellowish viscous oil. ^1^H-NMR (CDCl_3_) δ 2.86 (br.s, 8H, CH_2_N), 3.32 (t, *^3^J* = 3.9 Hz, 4H, CH_2_NPh), 3.61 (br.s, 8H, CH_2_O), 3.66 (s, 8H, CH_2_O), 3.70 (t, *^3^J* = 3.9 Hz, 4H, CH_2_O), 6.48–6.56 (m, 4H, H4(Ph), H6(Ph)), 7.07 (t, *^3^J* = 7.6 Hz, 2H, H5(Ph)), 7.12 (br.s, 2H, H2(Ph)), two NH protons were not assigned. ^13^C-NMR (CDCl_3_) δ 43.7 (2C, CH_2_NPh), 54.6 (4C, CH_2_N), 60.4 (2C, PhCH_2_N), 68.7 (4C, CH_2_O), 69.5 (2C, CH_2_O), 70.3 (2C, CH_2_O), 70.8 (2C, CH_2_O), 111.6 (2C, CH(Ph)), 114.6 (2C, CH(Ph)), 117.8 (2C, CH(Ph)), 128.9 (2C, C5(Ph)), 149.2 (2C, C3(Ph)), two quaternary carbon atoms C1(Ph) were not assigned. HRMS (MALDI-TOF): C_30_H_47_N_4_O_5_ (M+H)^+^ calcd.; 543.3546 observed; 543.3598.

*12,15,18,32,37-Pentaoxa-1,8,22,29-tetraazatetracyclo[27.5.5.1^3,7^.1^23,27^]hentetraconta-3(41),4,6,23 (40),24,26-hexaene* (**11k**). Obtained from compound **4** (0.2 mmol, 102 mg), trioxadiamine **10k** (0.2 mmol, 44 mg) in the presence of Pd(dba)_2_ (9 mg, 8 mol%), BINAP (11 mg, 9 mol%), NaO*t-*Bu (0.6 mmol, 57 mg) in abs. dioxane (10 mL). Eluent CH_2_Cl_2_–MeOH–NH_3_(aq) = 100:20:2. Yield 22 mg (19%), yellowish viscous oil. ^1^H-NMR (CDCl_3_) δ 1.84 (quintet, *^3^J* = 6.0 Hz, 4H, CH_2_CH_2_CH_2_), 2.74 (br.s, 8H, CH_2_N), 3.23 (t, *^3^J* = 6.4 Hz, 4H, CH_2_NPh), 3.51–3.63 (m, 16H, CH_2_O, PhCH_2_N), 3.64–3.69 (m, 4H, CH_2_O), 6.48 (d, *^3^J* = 8.1 Hz, 2H, H4(Ph) or H6(Ph)), 6.58 (d, *^3^J* = 7.2 Hz, 2H, H6(Ph) or H4(Ph)), 6.90 (s, 2H, H2(Ph)), 7.07 (t, *^3^J* = 7.6 Hz, 2H, H5(Ph)), two NH protons were not assigned. ^13^C-NMR (CDCl_3_) δ 29.0 (2C, CH_2_CH_2_CH_2_), 41.7 (2C, CH_2_NPh), 54.9 (4C, CH_2_N), 61.1 (2C, PhCH_2_N), 69.6 (4C, CH_2_O), 69.7 (2C, CH_2_O), 70.2 (2C, CH_2_O), 70.6 (2C, CH_2_O), 111.0 (2C, CH(Ph), 113.5 (2C, CH(Ph)), 117.3 (2C, CH(Ph)), 128.7 (2C, C5(Ph)), 140.5 (2C, C1(Ph)), 148.9 (2C, C3(Ph)). HRMS (MALDI-TOF): C_32_H_51_N_4_O_5_ (M+H)^+^ calcd.; 571.3859 observed; 571.3832.

*Cyclodimer* **13k**. Obtained as the second product in the synthesis of macrobicycle **11k**. Eluent CH_2_Cl_2_–MeOH–NH_3_(aq) = 100:20:2. Yield 27 mg (23%) of a yellowish glassy compound. ^1^H-NMR (CDCl_3_) δ 1.84 (quintet, *^3^J* = 6.0 Hz, 8H, CH_2_CH_2_CH_2_), 2.73 (br.s, 16H, CH_2_N), 3.20 (t, *^3^J* = 6.3 Hz, 8H, CH_2_NPh), 3.50–3.70 (m, 48H, CH_2_O, PhCH_2_N), 6.43–6.46 (m, 8H, H4(Ph), H6(Ph)), 6.63 (s, 4H, H2(Ph)), 7.07 (t, *^3^J* = 7.6 Hz, 4H, H5(Ph)), four NH protons were not assigned. ^13^C-NMR (CDCl_3_) δ 29.1 (4C, CH_2_CH_2_CH_2_), 41.6 (4C, CH_2_NPh), 57.7 (8C, CH_2_N), 61.2 (4C, PhCH_2_N), 69.4 (4C, CH_2_O), 69.6 (8C, CH_2_O), 70.2 (4C, CH_2_O), 70.6 (4C, CH_2_O), 111.1 (4C, CH(Ph)), 113.5 (4C, CH(Ph)), 117.8 (4C, CH(Ph)), 128.9 (4C, C5(Ph)), 140.3 (4C, C1(Ph)), 148.5 (4C, C3(Ph)). MS (MALDI-TOF): C_64_H_101_N_8_O_10_ (M+H)^+^ calcd.; 1141.76 observed; 1141.74.

*10,13,25,30-Tetraoxa-1,7,16,22-tetraazatetracyclo[20.5.5.2^3,6^.2^17,20^]hexatriaconta-3,5,17,19,33,35-hexaene* (**12h**). Obtained from compound **5** (0.2 mmol, 102 mg), dioxadiamine **10h** (0.2 mmol, 30 mg) in the presence of Pd(dba)_2_ (18 mg, 16 mol%), BINAP (22 mg, 18 mol%), NaO*t-*Bu (0.6 mmol, 57 mg) in abs. dioxane (10 mL). Eluent CH_2_Cl_2_–MeOH–NH_3_(aq) = 100:20:2. Yield 30 mg (30%), of a yellowish viscous oil. ^1^H-NMR (CDCl_3_) δ 2.73 (br.s, 8H, CH_2_N), 3.30 (t, *^3^J* = 5.1 Hz, 4H, CH_2_NPh), 3.52 (s, 4H, PhCH_2_N), 3.58 (br.s, 8H, CH_2_O), 3.66 (s, 4H, CH_2_O), 3.73 (t, *^3^J* = 5.1 Hz, 4H, CH_2_O), 4.07 (br.s, 2H, NH), 6.58 (d, *^3^J_obs_* = 8.3 Hz, 4H, H3(Ph)), 7.24 (d, *^3^J_obs_* = 8.3 Hz, 4H, H2(Ph)). ^13^C-NMR (CDCl_3_) δ 43.9 (2C, CH_2_NPh), 55.3 (4C, CH_2_N), 60.1 (2C, PhCH_2_N), 69.3 (2C, CH_2_O), 70.1 (6C, CH_2_O), 113.1 (4C, C3(Ph)), 129.7 (4C, C2(Ph)), 132.1 (2C, C1(Ph)), 147.2 (2C, C4(Ph)). HRMS (MALDI-TOF): C_28_H_43_N_4_O_4_ (M+H)^+^ calcd.; 499.3284 observed; 499.3318.

*Cyclodimer* **14h**. Obtained as the second product in the synthesis of macrobicycle **12h**. Eluent CH_2_Cl_2_–MeOH–NH_3_(aq) = 100:20:2. Yield 27 mg (27%) of a yellowish glassy compound. ^1^H-NMR (CDCl_3_) δ 2.71 (br.s, 16H, CH_2_N), 3.28 (t, *^3^J* = 4.4 Hz, 8H, CH_2_NPh), 3.51–3.61 (m, 24H, CH_2_O, PhCH_2_N), 3.64 (s, 8H, CH_2_O), 3.70 (t, *^3^J* = 4.4 Hz, 8H, CH_2_O), 4.06 (br.s, 4H, NH), 6.56 (d, *^3^J_obs_* = 8.2 Hz, 8H, H3(Ph)), 7.16 (d, *^3^J_obs_* = 8.2 Hz, 8H, H2(Ph)). ^13^C-NMR (CDCl_3_) δ 43.5 (4C, CH_2_NPh), 54.7 (8C, CH_2_N), 60.5 (4C, PhCH_2_N), 69.3 (8C, CH_2_O), 69.7 (4C, CH_2_O), 70.2 (4C, CH_2_O), 112.8 (8C, C3 (Ph)), 130.1 (8C, C2(Ph)), 131.9 (4C, C1(Ph)), 147.1 (4C, C4(Ph)). MS (MALDI-TOF): C_56_H_85_N_8_O_8_ (M+H)^+^ calcd.; 997.65 observed; 997.66.

*10,13,16,28,33-Pentaoxa-1,7,19,25-tetraazatetracyclo[23.5.5.2^3,6^.2^20,23^]nonatriaconta-3,5,20,22,36, 38-hexaene* (**12j**). Obtained from compound **5** (0.2 mmol, 102 mg), trioxadiamine **10j** (0.2 mmol, 38 mg) in the presence of Pd(dba)_2_ (9 mg, 8 mol%), BINAP (11 mg, 9 mol%), NaO*t-*Bu (0.6 mmol, 57 mg) in abs. dioxane (10 mL). Eluent CH_2_Cl_2_–MeOH–NH_3_(aq) = 100:20:2. Yield 26 mg (20%), of a yellowish viscous oil. ^1^H-NMR (CDCl_3_) δ 2.71 (t, *^3^J* = 4.0 Hz, 8H, CH_2_N), 3.31 (t, *^3^J* = 5.1 Hz, 4H, CH_2_NPh), 3.50 (s, 4H, PhCH_2_N), 3.60 (t, *^3^J* = 4.0 Hz, 8H, CH_2_O), 3.67 (s, 8H, CH_2_O), 3.72 (t, *^3^J* = 5.1 Hz, 4H, CH_2_O), 6.63 (d, *^3^J_obs_* = 8.3 Hz, 4H, H3(Ph)), 7.30 (d, *^3^J_obs_* = 8.3 Hz, 4H, H2(Ph)), two NH protons were not assigned. ^13^C-NMR (CDCl_3_) δ 43.7 (2C, CH_2_NPh), 55.2 (4C, CH_2_N), 60.0 (2C, PhCH_2_N), 69.6 (2C, CH_2_O), 70.1 (4C, CH_2_O), 70.4 (2C, CH_2_O), 70.8 (2C, CH_2_O), 112.9 (4C, C3(Ph)), 129.0 (2C, C1(Ph)), 129.6 (4C, C2(Ph)), 147.1 (2C, C4(Ph)). HRMS (MALDI-TOF): C_30_H_47_N_4_O_5_ (M+H)^+^ calcd.; 543.3546 observed; 543.3511.

*11,14,17,30,35-Pentaoxa-1,7,21,27-tetraazatetracyclo[25.5.5.2^3,6^.2^22,25^]hentetraconta-3,5,22,24,38, 40-hexaene* (**12k**). Obtained from compound **5** (0.2 mmol, 102 mg), trioxadiamine **10k** (0.2 mmol, 44 mg) in the presence of Pd(dba)_2_ (9 mg, 8 mol%), BINAP (11 mg, 9 mol%), NaO*t-*Bu (0.6 mmol, 57 mg) in abs. dioxane (10 mL). Eluent CH_2_Cl_2_–MeOH–NH_3_(aq) = 100:20:2. Yield 18 mg (15%), of a yellowish viscous oil. ^1^H-NMR (CDCl_3_) δ 1.89 (quintet, *^3^J* = 5.9 Hz, 4H, CH_2_CH_2_CH_2_), 2.71 (t, *^3^J* = 4.0 Hz, 8H, CH_2_N), 3.25 (t, *^3^J* = 6.3 Hz, 4H, CH_2_NPh), 3.51 (s, 4H, PhCH_2_N), 3.57 (t, *^3^J* = 4.0 Hz, 8H, CH_2_O), 3.60–3.65 (m, 4H, CH_2_O), 3.62 (t, *^3^J* = 5.2 Hz, 4H, CH_2_O), 3.68–3.72 (m, 4H, CH_2_O), 4.24 (br.s, 2H, NH), 6.57 (d, *^3^J_obs_* = 8.3 Hz, 4H, H3(Ph)), 7.24 (d, *^3^J_obs_* = 8.3 Hz, 4H, H2(Ph)). ^13^C-NMR (CDCl_3_) δ 28.9 (2C, CH_2_CH_2_CH_2_), 42.3 (2C, CH_2_NPh), 55.1 (4C, CH_2_N), 60.5 (2C, PhCH_2_N), 69.8 (4C, CH_2_O), 70.1 (2C, CH_2_O), 70.3 (2C, CH_2_O), 70.6 (2C, CH_2_O), 112.4 (4C, C3(Ph)), 130.0 (4C, C2(Ph)), 132.0 (2C, C1(Ph)), 147.6 (2C, C4(Ph)). HRMS (MALDI-TOF): C_32_H_51_N_4_O_5_ (M+H)^+^ calcd.; 571.3859 observed; 571.3890. 

*Cyclodimer* **14k**. Obtained as the second product in the synthesis of macrobicycle **12k**. Eluent CH_2_Cl_2_–MeOH–NH_3_(aq) = 100:20:3. Yield 20 mg (17%), of a yellowish glassy compound. ^1^H-NMR (CDCl_3_) δ 1.86 (quintet, *^3^J* = 5.3 Hz, 8H, CH_2_CH_2_CH_2_), 2.70 (br.s, 16H, CH_2_N), 3.19 (t, *^3^J* = 5.7 Hz, 8H, CH_2_NPh), 3.50–3.61 (m, 40H, CH_2_O, PhCH_2_N), 3.63–3.67 (m, 8H, CH_2_O), 3.85 (br.s, 4H, NH), 6.53 (d, *^3^J_obs_* = 8.3 Hz, 8H, H3(Ph)), 7.11 (d, *^3^J_obs_* = 8.3 Hz, 8H, H2(Ph)). ^13^C-NMR (CDCl_3_) δ 29.1 (4C, CH_2_CH_2_CH_2_), 41.8 (4C, CH_2_NPh), 54.4 (8C, CH_2_N), 60.6 (4C, PhCH_2_N), 69.4 (8C, CH_2_O), 69.7 (4C, CH_2_O), 70.2 (4C, CH_2_O), 70.6 (4C, CH_2_O), 112.4 (8C, C3(Ph)), 130.2 (8C, C2(Ph)), 130.4 (4C, C1(Ph)), 147.5 (4C, C4(Ph)). MS (MALDI-TOF): C_64_H_101_N_8_O_10_ (M+H)^+^ calcd.; 1141.76 observed; 1141.78.

*22,25,30-Trioxa-1,8,12,19-tetraazatetracyclo[17.8.5.1^3,7^.1^13,17^]tetratriaconta-3(34),4,6,13(33),14,16-hexaene* (**15а**). Obtained from compound **6** (0.25 mmol, 139 mg), diamine **10a** (0.25 mmol, 19 mg) in the presence of Pd(dba)_2_ (12 mg, 8 mol%), BINAP (14 mg, 9 mol%), *t*BuONa (0.75 mmol, 72 mg) in abs. dioxane (12 mL). Eluent CH_2_Cl_2_–MeOH = 3:1. Yield 22 mg (19%), of a yellowish glassy compound. ^1^H-NMR (CDCl_3_) δ 1.75 (br.s, 1Н, CH_2_CH_2_CH_2_), 2.06 (br.s, 1Н, CH_2_CH_2_CH_2_), 2.17 (d, *^2^J* = 13.3 Hz, 2H), 2.38 (dd, *^2^J* = 12.1 Hz, 2H), 2.72–2.96 (m, 4Н), 3.18–3.25 (m, 2Н), 3.26–3.32 (m, 4Н), 3.35-3.41 (m, 2Н), 3.47–3.78 (m, 8Н), 3.91 (d, *^3^J* = 11.6 Hz, 2H), 4.02 (t, *^3^J* = 9.0 Hz, 2H), 4.10 (br.s, 2Н, NH), 6.35 (d, *^3^J_obs_* = 6.9 Hz, 2H, H4(Ph) or H6(Ph)), 6.47 (d, *^3^J* = 7.8 Hz, 2H, H6(Ph) or H4(Ph)), 7.01 (t, *^3^J* = 7.8 Hz, 2H, H5(Ph)), 7.43 (br.s, 2Н, H2(Ph)). ^13^C-NMR (CDCl_3_) δ 28.9 (1C, CH_2_CH_2_CH_2_), 41.7 (2C, CH_2_NPh), 53.2 (2C, CH_2_N), 54.7 (2C, CH_2_N), 60.7 (2C, PhCH_2_N), 70.2 (2C, CH_2_O), 111.8 br (2C, CH(Ph)), 113.9 br (2C, CH(Ph)), 118.8 br (2C, CH(Ph)), 129.1 (2С, C5(Ph)), 148.7 (2C, C3(Ph)), two quaternary atoms С1(Ph) were not assigned due to a broad signal line; four СН_2_О carbon atoms give a very broad signal in the region 68–70 ppm). HRMS (MALDI-TOF): C_27_H_41_N_4_O_3_ (M+H)^+^ calcd.; 469.3178 observed; 469.3143.

*1,8,12,19,28,35,39,46-Octaazaheptacyclo[44.8.5.519,28.1^3,7^.1^13,17^.1^30,34^.1^40,44^]octahexaconta-3(68),4,6, 13(67),14,16,30(61),31,33,40(60),41,43-dodecaene* (**19а**). Obtained as the second product in the synthesis of macrobicycle **15a**. Eluent CH_2_Cl_2_–MeOH = 3:1. Yield 22 mg (19%) of a yellowish glassy compound. ^1^H-NMR (CDCl_3_) δ 1.84 (br.s, 4Н ССН_2_С), 2.71–2.96 (m, 16Н, CH_2_N), 3.29 (t, *^3^J* = 5.4 Hz, 8H, CH_2_NPh), 3.48–3.79 (m, 32Н, CH_2_O, PhCH_2_N), 6.49 (br.s, 4H, H4(Ph) or H6(Ph)), 6.55 (br.s, 4Н, H6(Ph) or H4(Ph)), 7.02–7.08 (m, 8Н, H2(Ph), H5(Ph)), NH protons were not assigned. ^13^C-NMR (CDCl_3_) δ 27.3 (2C, CCH_2_C), 42.9 (4C, CH_2_NPh), 53.2 (4C, CH_2_N), 54.7 (4C, CH_2_N), 61.9 (4C, PhCH_2_N), 67.3 (4C, CH_2_O), 67.7 (4C, CH_2_O), 69.4 (4C, CH_2_O), 110.7 (4C, CH(Ph)), 115.6 (4C, CH(Ph)), 118.2 (4C, CH(Ph)), 128.5 (4С, C5(Ph)), 137.7 (4C, C1(Ph)), 149.6 (4C, C3(Ph)). HRMS (MALDI-TOF): C_54_H_81_N_8_O_8_ (M+H)^+^ calcd.; 937.6279 observed; 937.6385.

*23,26,3,-Trioxa-1,8,13,20-tetraazatricyclo[18.8.5.1^3,7^.1^14,18^]pentatriaconta-3(35),4,6,14(34),15,17-hexaene* (**15b**). Obtained from compound **6** (0.25 mmol, 139 mg), diamine **10b** (0.25 mmol, 22 mg) in the presence of Pd(dba)_2_ (12 mg, 8 mol%), BINAP (14 mg, 9 mol%), NaO*t-*Bu (0.75 mmol, 72 mg) in abs. dioxane (12 mL). Eluent CH_2_Cl_2_–MeOH = 3:1. Yield 14 mg (12%) of a yellowish glassy compound. ^1^H-NMR (CDCl_3_) δ 1.83 (br.s, 4Н, CCH_2_CH_2_C), 2.85–3.08 (m, 4Н, CH_2_N), 3.15–3.22 (m, 4Н, CH_2_N), 3.40 (br.s, 4Н, CH_2_NPh), 3.53–3.88 (m, 16Н, CH_2_O, PhCH_2_N), 3.97 (br.s, 2Н, NH), 6.41 (br.s, 2Н, H4(Ph) or H6(Ph)), 6.49 (br.s, 2H, H6(Ph) pr H4(Ph)), 7.03 (t, *^3^J* = 7.6 Hz, 2H, H5(Ph)), 7.39 (br.s, 2Н, H2(Ph)). ^13^C-NMR (CDCl_3_) δ 26.6 (2C, CCH_2_CH_2_C), 43.5 br (2C, CH_2_NAr), 53.2 (2C, CH_2_N), 54.2 (2C, CH_2_N), 61.3 (2C, PhCH_2_N), 69.0 (2C, CH_2_O), 70.0 (4C, CH_2_O), 111.1 br (2C, CH(Ph)), 115.8 br (2C, CH(Ph)), 118.1 br (2C, CH(Ph)), 128.6 (2С, C5(Ph)), 148.8 (2C, C3(Ph)), two quaternary atoms С1(Ph) were not assigned due to a broad signal line. HRMS (MALDI-TOF): C_28_H_43_N_4_O_3_ (M+H)^+^ calcd.; 483.3335 observed; 483.3275.

*22,25,50,53,58,65-Hexaoxa-1,7,12,19,28,35,40,47-octaazaheptacyclo-[45.8.5.5^19,28^.2^3,6^.1^13,17^.1^30,34^.1^41,45^]octahexaconta-3,5,13(68),14,16,30(62),31,33,41(61),42,44,69-dodecaene* (**19b**). Obtained as the second product in the synthesis of macrobicycle **15b**. Eluent CH_2_Cl_2_–MeOH = 3:1. Yield 25 mg (21%) of a yellowish glassy compound. ^1^H-NMR (CDCl_3_) δ 1.71 (br.s, 8Н ССН_2_С), 2.45–3.08 (m, 16Н CH_2_N), 3.18 (t, *^3^J* = 5.5 Hz, 8H, CH_2_NPh), 3.52–3.82 (m, 32Н, CH_2_O, PhCH_2_N), 4.65 (br.s, 4Н, NH), 6.48 (d, *^3^J_obs_* = 7.2 Hz, 4H, H(Ph) or Н6(Ph)), 6.56 (br.s, 4Н, Н6(Ph) or H4(Ph)), 7.01 (br.s, 4Н, Н2(Ph)), 7.03 (t, *^3^J* = 7.6 Hz, 4H, H5(Ph)). ^13^C-NMR (CDCl_3_) δ 26.6 (4C, CCH_2_CH_2_C), 43.1 (4C, CH_2_NPh), 53.9 (4C, CH_2_N), 54.9 (4C, CH_2_N), 60.3 (4C, PhCH_2_N), 67.2 (4C, CH_2_O), 67.4 (4C, CH_2_O), 67.9 (4C, CH_2_O), 112.6 (4C, CH(Ph)), 112.9 (4C, CH(Ph)), 118.1 (4C, CH(Ph)), 128.9 (4С, C5(Ph)), 137.5 (4C, C1(Ph)), 148.6 (4C, C3(Ph)). HRMS (MALDI-TOF): C_56_H_85_N_8_O_6_ (M+H)^+^ calcd.; 965.6592 observed; 965.6511.

*29,32,37-Trioxa-1,8,19,26-tetraazatetracyclo[24.8.5.1^3,7^.1^20,24^]hentetraconta-3(41),4,6,20(40),21,23-hexaene* (**15c**). Obtained from compound **6** (0.25 mmol, 139 mg), diamine **10c** (0.25 mmol, 43 mg) in the presence of Pd(dba)_2_ (12 mg, 8 mol%), BINAP (14 mg, 9 mol%), NaO*t-*Bu (0.75 mmol, 72 mg) in abs. dioxane (12 mL). Eluent CH_2_Cl_2_–MeOH = 10:1. Yield 38 mg (27%) of a yellowish glassy compound. ^1^H-NMR (CDCl_3_) δ 1.10–1.37 (m, 12Н, CCH_2_C), 1.45–1.55 (m, 4H, CH_2_CNPh) 2.60–3.21 (m, 12Н, CH_2_N, CH_2_NPh), 3.58–3.83 (m, 16Н, CH_2_O, PhCH_2_N), 4.22 (br.s, 2Н, NH), 6.48 (d, *^3^J_obs_* = 6.7 Hz, 2H, H4(Ph)), 6.59 (d, *^3^J_obs_* = 5.7 Hz, 2H, H6(Ph)), 6.84 (br.s 2H, H2(Ph)), 7.05 (t, *^3^J* = 7.7 Hz, 2H, H5(Ph)). ^13^C-NMR (CDCl_3_) δ 26.1 (2C, CCH_2_C), 28.1 (2C, CCH_2_C), 28.2 (2C, CCH_2_C), 28.7 (2C, СCCH_2_C), 43.5 (2C, CH_2_NPh), 53.9 (2C, CH_2_N), 54.3 (2C, CH_2_N), 60.2 (2C, PhCH_2_N), 67.3 (2C, CH_2_O), 67.5 (2C, CH_2_O), 70.0 (2C, CH_2_O), 112.0 (2C, CH(Ph)), 114.0 (2C, CH(Ph)), 117.8 (2C, CH(Ph)), 129.2 (2С, C5(Ph)), 137.4 (2C, C1(Ph)), 148.9 (2C, C3(Ph)). HRMS (MALDI-TOF): C_34_H_55_N_4_O_3_ (M+H)^+^ calcd.; 567.4274 observed; 567.4225.

*29,32,63,66,71,78-Hexaoxa-1,8,19,26,35,42,53,60-octaazaheptacyclo-[58.8.5.5^26,35^.1^3,7^.1^20,24^.1^37,41^.1^54,58^]dooctaconta-3(82),4,6,20(81),21,23,37(75),38,40,54(74),55,57-dodecaene* (**19с**). Obtained as the second product in the synthesis of macrobicycle **15c**. Eluent CH_2_Cl_2_–MeOH = 3:1. Yield 21 mg (15%) of a yellowish glassy compound. ^1^H-NMR (CDCl_3_) δ 1.15–1.37 (m, 24Н, CCH_2_C), 1.50–1.59 (m, 8H, CH_2_CNAr), 2.67–2.92 (m, 16Н, CH_2_N), 3.02 (br.s, 8Н, CH_2_NPh), 3.50–3.75 (m, 32Н, CH_2_O, PhCH_2_N), 4.38 (br.s, 4Н, NH), 6.46 (d, *^3^J* = 8.1 Hz, 4H, H4(Ph)), 6.56 (br.s, 4H, H6(Ph)), 6.72 (br.s, 4Н, H2(Ph)), 7.05 (t, *^3^J* = 7.7 Hz, 4H, H5(Ph)). ^13^C-NMR (CDCl_3_) δ 27.1 (4C, CCH_2_C), 29.4 (12C, CCH_2_C), 43.9 (4C, CH_2_NPh), 53.3 (4C, CH_2_N), 53.9 (4C, CH_2_N), 60.2 (4C, PhCH_2_N), 67.0–70.0 (m, 12C, CH_2_O), 111.1 (4C, CH(Ph)), 114.2 (4C, CH(Ph)), 118.0 (4C, CH(Ph)), 129.0 (4С, C5(Ph)), 148.8 (4C, C3(Ph)), four quaternary C1(Ph) atoms were not assigned. HRMS (MALDI-TOF): C_68_H_109_N_8_O_6_ (M+H)^+^ calcd.; 1133.8470 observed; 1133.8562.

*26,29,34-Trioxa-1,8,12,16,23-pentaazatetracyclo[21.8.5.1^3,7^.1^17,21^]octatriaconta-3(38),4,6,17(37), 18,20-hexaene* (**15d**). Obtained from compound **6** (0.25 mmol, 139 mg), triamine **10d** (0.25 mmol, 33 mg) in the presence of Pd(dba)_2_ (12 mg, 8 mol%), BINAP (14 mg, 9 mol%), NaO*t-*Bu (0.75 mmol, 72 mg) in abs. dioxane (12 mL). Eluent CH_2_Cl_2_–MeOH–NH_3_(aq) = 100:20:3. Yield 47 mg (36%) of a yellow glassy compound. ^1^H-NMR (CDCl_3_) δ 1.92 (br.s, 4Н, ССН_2_С), 2.65–2.75 (m, 8Н, CH_2_N), 2.88 (br.s, 4Н СН_2_NHСН_2_), 3.21 (t, *^3^J* = 5.4 Hz, 4H, CH_2_NPh), 3.53 (s, 4Н, PhCH_2_N), 3.55–3.65 (m, 12H, CH_2_O), 5.10 (br.s, 2Н, PhNH), 6.43 (br.s, 2Н, H4(Ph) or H6(Ph)), 6.50 (d, *^3^J_obs_* = 6.7 Hz, 2H, H6(Ph) or H4(Ph)), 6.90 (br.s, 2Н, H2(Ph)), 7.03 (t, *^3^J* = 7.7 Hz, 2H, Н5(Ph)), one NH proton was not assigned. ^13^C-NMR (CDCl_3_) δ 26.4 (2C, CCH_2_C), 41.6 (2C, CH_2_NPh), 46.3 (2C, CH_2_NHCH_2_), 54.2 (2C, CH_2_N), 54.9 (2C, CH_2_N), 60.4 (2C, PhCH_2_N), 69.2 (4C, CH_2_O), 70.0 (2C, CH_2_O), 110.7 (2C, CH(Ph)), 113.6 (2C, CH(Ph)), 117.5 (2C, CH(Ph)), 128.9 (2С, C5(Ph)), 140.4 (2C, C1(Ph)), 148.2 (2C, C3(Ph)). HRMS (MALDI-TOF): C_30_H_48_N_5_O_3_ (M+H)^+^ calcd.; 526.3757 observed; 526.3798.

*26,29,57,60,65,72-Hexaoxa-1,8,12,16,23,32,39,43,47,54-decaazaheptacyclo-[52.8.5.5^23,32^.1^3,7^.1^17,21^.1^34,38^.1^48,52^]-hexaheptaconta-3(76),4,6,17(75),18,20,34(69),35,37,48(68),49,51-dodecaene* (**19d**). Obtained as the second product in the synthesis of macrobicycle **15d**. Eluent CH_2_Cl_2_–MeOH–NH_3_(aq) = 100:25:5. Yield 12 mg (9%) of a yellowish glassy compound. ^1^H-NMR (CDCl_3_) δ 1.80 (br.s, 8Н, ССН_2_С), 2.60–2.82 (m, 24Н, CH_2_N), 3.14 (br.s, 8Н, CH_2_NPh), 3.48–3.68 (m, 32H, CH_2_O, PhCH_2_N), 6.44 (br.s, 4Н, H4(Ph) or H6(Ph)), 6.55 (br.s 4H, H6(Ph) or H4(Ph)), 6.79 (br.s, 4Н, H2(Ph)), 7.05 (br.s, 4Н, Н5(Ph)), NH protons were not assigned. MS (MALDI-TOF): C_60_H_95_N_10_O_6_ (M+H)^+^ calcd.; 1051.74 observed; 1051.72.

*28,31,36-Trioxa-1,8,11,15,18,25-hexatetracyclo[23.8.5.1^3,7^.1^19,23^]tetraconta-3(40),4,6,19(39),20,22-hexaene* (**15е**). Obtained from compound **6** (0.25 mmol, 139 mg), tetraamine **10e** (0.25 mmol, 40 mg) in the presence of Pd(dba)_2_ (12 mg, 8 mol%), BINAP (14 mg, 9 mol%), NaO*t-*Bu (0.75 mmol, 72 mg) in abs. dioxane (12 mL). Eluent CH_2_Cl_2_–MeOH–NH_3_(aq) = 100:25:5. Yield 39 mg (28%) of a yellow glassy compound. ^1^H-NMR (CDCl_3_) δ 1.78 (quintet, *^3^J* = 5.5 Hz, 2H, ССН_2_С), 2.70 (t, *^3^J* = 5.5 Hz, 8H, СН_2_NHСН_2_), 2.82 (t, *^3^J* = 5.7 Hz, 4H, СН_2_N), 2.87 (t, *^3^J* = 5.2 Hz, 4H, CH_2_N), 3.31 (t, *^3^J* = 4.7 Hz, 4H, CH_2_NPh), 3.53 (s, 4H, PhCH_2_N), 3.56–3.66 (m, 12H, CH_2_O), 4.79 (br.s, 2Н, PhNH), 6.51 (d, *^3^J* = 7.7 Hz, 2H, H4(Ph) or H6(Ph)), 6.54 (dd, *^3^J* = 7.8 Hz, *^4^J* = 1.5 Hz, 2H, H6(Ph) or H4(Ph)), 6.96 (br.s, 2Н, H2(Ph)), 7.03 (t, *^3^J* = 7.8 Hz, 2H, Н5(Ph)), two NH protons were not assigned. ^13^C-NMR (CDCl_3_) δ 25.9 (1C, CCH_2_C), 42.5 (2C, CH_2_NPh), 47.7 (2C, CH_2_NHCH_2_), 48.8 (2C, CH_2_NHCH_2_), 54.9 (2C, CH_2_N), 55.3 (2C, CH_2_N), 60.5 (2C, PhCH_2_N), 69.3 (2C, CH_2_O), 69.6 (2C, CH_2_O), 70.1 (2C, CH_2_O), 110.8 (2C, CH(Ph)), 113.7 (2C, CH(Ph)), 117.6 (2C, CH(Ph)), 128.8 (2С, C5(Ph)), 140.9 (2C, C1(Ph)), 148.3 (2C, C3(Ph)). HRMS (MALDI-TOF): C_31_H_51_N_6_O_3_ (M+H)^+^ calcd.; 555.4022 observed; 555.3979.

*29,32,37-Trioxa-1,8,12,15,19,26-hexaazatetracyclo[24.8.5.1^3,7^.1^20,24^]hentetraconta-3(41),4,6,20(40), 21,23-hexaene* (**15f**). Obtained from compound **6** (0.25 mmol, 139 mg), tetraamine **10f** (0.25 mmol, 44 mg) in the presence of Pd(dba)_2_ (12 mg, 8 mol%), BINAP (14 mg, 9 mol%), NaO*t-*Bu (0.75 mmol, 72 mg) in abs. dioxane (12 mL). Eluent CH_2_Cl_2_–MeOH–NH_3_(aq) = 100:25:5. Yield 47mg (33%) of a yellow glassy compound. ^1^H-NMR (CDCl_3_) δ 1.74 (quintet, *^3^J* = 6.3 Hz, 4H, ССН_2_С), 2.55–2.81 (m, 16Н, CH_2_N), 3.15 (t, *^3^J* = 6.3 Hz, 4H, CH_2_NPh), 3.51–3.66 (m, 16H, CH_2_O, PhCH_2_N), 6.43 (d, *^3^J* = 7.8 Hz, 2H, H4(Ph) or H6(Ph)), 6.54 (d, *^3^J* = 7.3 Hz, 2H, H6(Ph) or H4(Ph)), 6.90 (br.s, 2Н, H2(Ph)), 7.04 (t, *^3^J* = 7.7 Hz, 2H, Н5(Ph)) NH protons were not assigned. ^13^C-NMR (CDCl_3_) δ 28.8 (2C, CCH_2_C), 42.5 (2C, CH_2_NPh), 47.7 (2C, CH_2_NHCH_2_), 48.3 (2C, CH_2_NHCH_2_), 54.5 (2C, CH_2_N), 54.9 (2C, CH_2_N), 60.4 (2C, PhCH_2_N), 64.9 (4C, CH_2_O), 70.1 (2C, CH_2_O), 110.4 (2C, CH(Ph)), 113.9 (2C, CH(Ph)), 117.3 (2C, CH(Ph)), 128.8 (2С, C5(Ph)), 140.5 (2C, C1(Ph)), 148.7 (2C, C3(Ph)). HRMS (MALDI-TOF): C_32_H_53_N_6_O_3_ (M+H)^+^ calcd.; 569.4179 observed; 569.4142.

*30,33,38-Trioxa-1,8,12,16,20,27-hexaazatetracyclo[25.8.5.1^3,7^.1^21,25^]dodecatetraconta-3(42),4,6,21 (41),22,24-hexaene* (**15g**). Obtained from compound **6** (0.25 mmol, 139 mg), tetraamine **10g** (0.25 mmol, 47 mg) in the presence of Pd(dba)_2_ (12 mg, 8 mol%), BINAP (14 mg, 9 mol%), NaO*t-*Bu (0.75 mmol, 72 mg) in abs. dioxane (12 mL). Eluent CH_2_Cl_2_–MeOH–NH_3_(aq) = 100:25:5. Yield 34 mg (24%) of a yellow glassy compound. ^1^H-NMR (CDCl_3_) δ 1.74 (quintet, *^3^J* = 7.3 Hz, 2H, ССН_2_С), 1.78 (quintet, *^3^J* = 5.3 Hz, 4H, ССН_2_С), 2.67–2.76 (m, 12Н, CH_2_N, CH_2_NHCH_2_), 2.79 (t, *^3^J* = 5.6 Hz, 4H, CH_2_N), 3.12 (t, *^3^J* = 6.3 Hz, 4H, CH_2_NPh), 3.53–3.65 (m, 16H, CH_2_O, PhCH_2_N), 4.27 (br.s, 2Н, PhNH), 6.46 (dd, *^3^J* = 7.7 Hz, *^4^J* = 1.8 Hz, 2H, H4(Ph) or H6(Ph)), 6.58 (d, *^3^J* = 7.7 Hz, 2H, H6(Ph) or H4(Ph)), 6.89 (br.s, 2Н, H2(Ph)), 7.05 (t, *^3^J* = 7.7 Hz, 2H, Н5(Ph), two NH protons were not assigned. ^13^C-NMR (CDCl_3_) δ 27.5 (1C, CCH_2_C), 28.4 (2C, CCH_2_C), 42.4 (2C, CH_2_NPh), 47.9 (2C, CH_2_NHCH_2_), 49.2 (2C, CH_2_NHCH_2_), 54.7 (2C, CH_2_N), 55.0 (2C, CH_2_N), 60.5 (2C, PhCH_2_N), 69.5 (4C, CH_2_O), 70.2 (2C, CH_2_O), 110.7 (2C, CH(Ph)), 113.6 (2C, CH(Ph)), 117.5 (2C, CH(Ph)), 128.8 (2С, C5(Ph)), 140.9 (2C, C1(Ph)), 148.7 (2C, C3(Ph)). HRMS (MALDI-TOF): C_33_H_55_N_6_O_3_ (M+H)^+^ calcd.; 583.4335 observed; 583.4390.

*11,14,27,30,35-Pentaoxa-1,8,17,24-tetraazatetracyclo[22.8.5.1^3,7^.1^18,22^]nonatriaconta-3(39),4,6,18 (38),19,21-hexaene* (**15h**). Obtained from compound **6** (0.25 mmol, 139 mg), dioxadiamine **10h** (0.25 mmol, 37 mg) in the presence of Pd(dba)_2_ (12 mg, 8 mol%), BINAP (14 mg, 9 mol%), NaO*t-*Bu (0.75 mmol, 72 mg) in abs. dioxane (12 mL). Eluent CH_2_Cl_2_–MeOH = 10:1. Yield 27 mg (20%) of a yellowish glassy compound. ^1^H-NMR (CDCl_3_) δ 2.61–3.15 (m, 8Н, CH_2_N), 3.30 (br.s, 4Н, CH_2_NPh), 3.50–3.75 (m, 24Н, CH_2_O, PhCH_2_N), 6.53 (dd, *^3^J* = 8.1 Hz, *^4^J* = 1.6 Hz, 2H, H4(Ph) or H6(Ph)), 6.58 (br.s, 2H, H6(Ph) or H4(Ph)), 7.06 (t, *^3^J* = 7.8 Hz, 2H, H5(Ph)), 7.19 (br.s, 2H, H2(Ph)), NH protons were not assigned. ^13^C-NMR (CDCl_3_) δ 43.9 (2C CH_2_NPh), 53.4 (2C, CH_2_N), 54.7 (2C, CH_2_N), 61.0 (2C, PhCH_2_N), 67.4 (2C, CH_2_O), 67.8 (2C, CH_2_O), 69.3 (4C, CH_2_O), 70.2 (2C, CH_2_O), 111.1 (2C, CH(Ph)), 116.3 (2C, CH(Ph)), 118.9 (2C, CH(Ph)), 128.9 (2С, C5(Ph)), 137.8 (2C, C1(Ph)), 149.1 (2C, C3(Ph)). HRMS (MALDI-TOF): C_30_H_47_N_4_O_5_ (M+H)^+^ calcd.; 543.3543 observed; 543.3588.

*10,13,26,29,42,45,58,61,66,73-Decaoxa-1,7,16,23,32,39,48,55-octaazaheptacyclo-[53.8.5.5.5.32,32.2^3,6^.1^17,21^.1^34,38^.1^49,53^]-ocatheptaconta-3,5,17(76),18,20,34(70),35,37,49(69),50,52,77-dodecaene* (**19h**). Obtained as the second product in the synthesis of macrobicycle **15h**. Eluent CH_2_Cl_2_–MeOH = 3:1. Yield 14 mg (10%) of a yellowish glassy compound. ^1^H-NMR (DMSO-*d_6_*, 363K) δ 2.84 (br.s, 16Н, CH_2_N), 3.21 (t, *^3^J* = 5.6 Hz, 8H, CH_2_NPh), 3.52-3.68 (m, 48Н, CH_2_O, PhCH_2_N), 6.50 (d, *^3^J* = 8.1 Hz, 4H, H4(Ph) or H6(Ph)), 6.52 (d, *^3^J* = 8.3 Hz, 4H, H6(Ph) or H4(Ph)), 6.72 (br.s, 4Н, H2(Ph)), 6.99 (t, *^3^J* = 7.8 Hz, 4H, H5(Ph)), NH protons were not assigned. ^13^C-NMR (DMSO-*d_6_*, 363K) δ 42.7 (4C, CH_2_NPh), 53.5 (4C, CH_2_N), 53.9 (4C, CH_2_N), 59.6 (4C, PhCH_2_N), 67.5 (4C, CH_2_O), 68.0 (4C, CH_2_O), 68.9 (4C, CH_2_O), 69.5 (8C, CH_2_O), 111.7 (4C, CH(Ph)), 112.2 (4C, CH(Ph)), 116.5 (4C, CH(Ph)), 128.1 (4C, C5(Ph)), 148.3 (4С, C3(Ph)), four quaternary C1(Ph) atoms were not assigned. HRMS (MALDI-TOF): C_60_H_93_N_8_O_10_ (M+H)^+^ calcd.; 1085.7014 observed; 1085.7086.

*11,16,29,32,37-Pentaoxa-1,8,19,26-tetraazatetracyclo[24.8.5.1^3,7^.1^20,24^]hentetraconta-3(41),4,6,20 (40),21,23-hexaene* (**15i**). Obtained from compound **6** (0.25 mmol, 139 mg), dioxadiamine **10i** (0.25 mmol, 51 mg) in the presence of Pd(dba)_2_ (12 mg, 8 mol%), BINAP (14 mg, 9 mol%), NaO*t-*Bu (0.75 mmol, 72 mg) in abs. dioxane (12 mL). Eluent CH_2_Cl_2_–MeOH = 10:1. Yield 56 mg (37%) as a yellowish glassy compound. ^1^H-NMR (CDCl_3_) δ 1.57–1.67 (m, 4H, ССH_2_CH_2_C), 1.80 (quintet, *^3^J* = 5.3 Hz, 4H, NCCH_2_CN), 2.57–3.16 (m, 12Н, CH_2_N), 3.36-3.43 (m, 4Н, CH_2_O), 3.47 (t, *^3^J* = 4.7 Hz, 4H, CH_2_O), 3.54–3.75 (m, 16Н, CH_2_O, PhCH_2_N), 6.47 (d, *^3^J_obs_* = 7.1 Hz, 4H, H4(Ph), H6(Ph)), 6.96 (br.s, 2Н, H2(Ph)), 7.05 (t, *^3^J* = 7.7 Hz, 2H, H5(Ph)), NH protons were not assigned. ^13^C-NMR (CDCl_3_) δ 26.4 (2C, ССH_2_CH_2_C), 29.1 (2C, NССH_2_N), 41.7 (2C, CH_2_NPh), 53.0 (2C, CH_2_N), 54.3 (2C, CH_2_N), 60.2 (2C, PhCH_2_N), 67.2 (2C, CH_2_O), 67.5 (2C, CH_2_O), 69.0 (2C, CH_2_O), 69.2 (2C, CH_2_O), 70.5 (2C, CH_2_O), 110.1 (2C, CH(Ph)), 115.3 (2C, CH(Ph)), 118.2 (2C, CH(Ph)), 128.9 (2С, C5(Ph)), 137.4 (2C, C1(Ph)), 149.3 (2C, C3(Ph)). HRMS (MALDI-TOF): C_34_H_55_N_4_O_5_ (M+H)^+^ calcd.; 599.4172 observed; 599.4130.

*12,15,18,32,35,40-Hexaoxa-1,8,22,29-tetraazatetracyclo[27.8.5.1^3,7^.1^23,27^]tetratetraconta-3(44),4,6,23 (43),24,26-hexaene* (**15k**). Obtained from compound **6** (0.25 mmol, 139 mg), trioxadiamine **10k** (0.25 mmol, 51 mg) in the presence of Pd(dba)_2_ (12 mg, 8 mol%), BINAP (14 mg, 9 mol%), NaO*t-*Bu (0.75 mmol, 72 mg) in abs. dioxane (12 mL). Eluent CH_2_Cl_2_–MeOH = 10:1. Yield 58 mg (38%) of a yellowish glassy compound. ^1^H-NMR (CDCl_3_) δ 1.82 (quintet, *^3^J* = 6.1 Hz, 4H, ССН_2_С), 2.74 (t, *^3^J* = 4.7 Hz, 4H, CH_2_N), 2.77 (t, *^3^J* = 4.7 Hz, 4H, СН_2_N), 3.18 (t, *^3^J* = 6.3 Hz, 4H, CH_2_NPh), 3.57 (t, *^3^J* = 5.8 Hz, 4H, CH_2_O), 3.57–3.67 (m, 24H, CH_2_O, PhCH_2_N), 4.10 (br.s, 2Н, NH), 6.45 (d, *^3^J* = 7.7 Hz, 2H, H4(Ph) or H6(Ph)), 6.55 (d, *^3^J* = 7.3 Hz, 2H, H6(Ph) or H4(Ph)), 6.83 (br.s, 2Н, H2(Ph)), 7.04 (t, *^3^J* = 7.8 Hz, 2H, Н5(Ph)). ^13^C-NMR (CDCl_3_) δ 28.9 (2C, CCH_2_C), 41.5 (2C, CH_2_NPh), 53.0 (2C, CH_2_N), 54.4 (2C, CH_2_N), 60.3 (2C, PhCH_2_N), 67.2 (2C, CH_2_O), 67.6 (2C, CH_2_O), 69.0 (2C, CH_2_O), 69.6 (2C, CH_2_O), 70.0 (2C, CH_2_O), 70.4 (2C, CH_2_O), 110.4 (2C, CH(Ph)), 115.2 (2C, CH(Ph)), 118.3 (2C, CH(Ph)), 128.8 (2С, C5(Ph)), 137.3 (2C, C1(Ph)), 149.4 (2C, C3(Ph)). HRMS (MALDI-TOF): C_34_H_55_N_4_O_6_ (M+H)^+^ calcd.; 615.4121 observed; 615.4157.

*12,15,18,32,35,49,52,55,69,72,77,84-Dodecaoxa-1,8,22,29,38,45,59,66-octaazaheptacyclo-[64.8.5.5^29,38^.1^3,7^.1^23,27^.1^40,44^.1^60,64^]octaoctaconta-3(88),4,6,23(87),24,26,40(81),41,43,60(80),61,63-dodeca-ene* (**19k**). Obtained as the second product in the synthesis of macrobicycle **15k**. Eluent CH_2_Cl_2_–MeOH = 3:1. Yield 37 mg (24%) of a yellowish glassy compound. ^1^H-NMR (CDCl_3_) δ 1.84 (quintet, *^3^J* = 5.8 Hz, 8H, ССН_2_С), 2.75 (t, *^3^J* = 4.8 Hz, 8H, CH_2_N), 2.78 (t, *^3^J* = 4.8 Hz, 8H, СН_2_N), 3.18 (t, *^3^J* = 5.9 Hz, 8H, CH_2_NPh), 3.53-3.68 (m, 56H, CH_2_O, PhCH_2_N), 4.06 (br.s, 4Н, NH), 6.44 (d, *^3^J* = 7.6 Hz, 4H, H4(Ph) or H6(Ph)), 6.59 (d, *^3^J* = 7.2 Hz, 4H, H6(Ph) or H4(Ph)), 6.69 (br.s, 4Н, H2(Ph)), 7.05 (t, *^3^J* = 7.7 Hz, 4H, Н5(Ph)). ^13^C-NMR (CDCl_3_) δ 29.0 (4C, CCH_2_C), 41.4 (4C, CH_2_NPh), 54.2 (8C, CH_2_N), 60.5 (4C, PhCH_2_N), 69.3 (4C, CH_2_O), 69.4 (4C, CH_2_O), 69.9 (4C, CH_2_O), 70.0 (4C, CH_2_O), 70.2 (4C, CH_2_O), 70.4 (4C, CH_2_O), 110.6 (4C, CH(Ph)), 113.2 (4C, CH(Ph)), 117.2 (4C, CH(Ph)), 128.7 (4С, C5(Ph)), 140.3 (4C, C1(Ph)), 148.5 (4C, C3(Ph)). MS (MALDI-TOF): C_68_H_109_N_8_O_6_ (M+H)^+^ calcd.; 1229.82 observed; 1229.84.

*10,13,25,28,33-Pentaoxa-1,7,16,22-tetraazatetracyclo[20.8.5.2^3,6^.2^17,20^]nonatriaconta-3,5,17,19,36, 38-hexaene* (**16h**). Obtained from compound **7** (0.5 mmol, 278 mg), dioxadiamine **10h** (0.5 mmol, 74 mg) in the presence of Pd(dba)_2_ (46 mg, 16 mol%), BINAP (56 mg, 18 mol%), NaO*t-*Bu (1.5 mmol, 144 mg) in abs. Dioxane (25 ml). Eluent CH_2_Cl_2_–MeOH = 5:1, CH_2_Cl_2_–MeOH–NH_3_(aq) = 100:20:1. Yield 49 mg (18%) as a yellow glassy compound. ^1^H-NMR (CDCl_3_) δ 2.69–2.86 (m, 8H, CH_2_N), 3.27 (t, *^3^J* = 4.5 Hz, 4H, CH_2_NPh), 3.51–3.70 (m, 24H, CH_2_O, PhCH_2_N), 6.54 (d, *^3^J_obs_* = 7.0 Hz, 4H, H3(Ph), H3'(Ph)), 7.09 (d, *^3^J_obs_* = 7.5 Hz, 4H, H2(Ph), H2'(Ph)), NH protons were not assigned. ^13^C-NMR (CDCl_3_) δ 43.4 (2C, CH_2_NPh), 52.8 (2C, CH_2_N), 54.8 (2C, CH_2_N), 60.1 (2C, PhCH_2_N), 67.0 (2C, CH_2_O), 69.0–69.9 (m, 8C, CH_2_O), 113.0 (4C, C3(Ph), C3'(Ph)), 126.0 (2C, C1(Ph)), 131.4 (4C, C2(Ph), C2'(Ph)), 148.3 (2C, C4(Ph)). HRMS (MALDI-TOF): C_30_H_47_N_4_O_5_ (M+H)^+^ calcd.; 543.3546 observed; 543.3577.

*10,13,25,40,43,55,58,63,72-Decaoxa-1,7,16,22,31,37,46,52-octaazaheptacyclo-[50.8.5.5^22,31^.2^3,6^.2^17,20^.2^33,36^.2^47,50^]octaheptaconta-3,5,17,19,33,35,47,49,66,68,75,77-dodecaene* (**20h**). Obtained as the second product in the synthesis of macrobicycle **16h**. Eluent CH_2_Cl_2_–MeOH–NH_3_(aq) = 100:20:1–100:20:3. Yield 85 mg (31%) of a yellow glassy compound. ^1^H-NMR (CDCl_3_) δ 2.59 (t, *^3^J* = 5.0 Hz, 8H, CH_2_N), 2.67 (t, *^3^J* = 4.6 Hz, 8H, CH_2_N), 3.27 (t, *^3^J* = 4.5 Hz, 8H, CH_2_NPh), 3.47 (br.s, 8H, CH_2_O), 3.51–3.64 (m, 16H, CH_2_O), 3.66 (s, 8H, CH_2_O or PhCH_2_N), 3.68 (s, 8H, PhCH_2_N or CH_2_O), 3.74 (t, *^3^J* = 5.0 Hz, 8H, CH_2_O), 4.04 (br.s, 4H, NH), 6.51 (d, *^3^J_obs_* = 8.1 Hz, 8H, H3(Ph), H3'(Ph)), 7.16 (d, *^3^J_obs_* = 8.1 Hz, 8H, H2(Ph), H2'(Ph)). ^13^C-NMR (CDCl_3_) δ 43.6 (4C, CH_2_NPh), 52.2 (4C, CH_2_N), 55.3 (4C, CH_2_N), 61.2 (4C, PhCH_2_N), 67.0–69.9 (m, 20C), 113.3 (8C, C3(Ph), C3'(Ph)), 125.0 (4C, C1(Ph)), 131.4 (8C, C2(Ph), C2'(Ph)), 147.6 (4C, C4(Ph)). HRMS (MALDI-TOF): C_60_H_93_N_8_O_10_ (M+H)^+^ calcd.; 1085.7014 observed; 1085.6952.

*11,16,29,32,37-Pentaoxa-1,7,20,26-tetraazatetracyclo[24.8.5.2^3,6^.2^21,24^]tritetraconta-3,5,21,23,42-hexaene* (**16i**). Obtained from compound **7** (0.25 mmol, 139 mg), dioxadiamine **10i** (0.25 mmol, 51 mg) in the presence of Pd(dba)_2_ (23 mg, 16 mol%), BINAP (28 mg, 18 mol%), NaO*t-*Bu (0.75 mmol, 72 mg) in abs. dioxane (12 mL). Eluent CH_2_Cl_2_–MeOH = 5:1. Yield 15 mg (10%) as a yellow glassy compound. ^1^H-NMR (CDCl_3_) δ 1.65 (br.s, 4H, CCH_2_CH_2_C), 1.86 (quintet, *^3^J* = 5.7 Hz, 4H, NCCH_2_CN), 2.58–3.10 (m, 8H, CH_2_N), 3.20 (t, *^3^J* = 5.7 Hz, 4H, CH_2_NPh), 3.37–3.45 (m, 4H, CH_2_O), 3.47–3.72 (m, 16H, CH_2_O, PhCH_2_N), 3.78-3.93 (m, 4H, CH_2_O), 6.51 (d, *^3^J_obs_* = 8.1 Hz, 4H, H3(Ph), H3'(Ph)), 7.21 (d, *^3^J_obs_* = 8.1 Hz, 4H, H2(Ph), H2'(Ph)), NH protons were not assigned. ^13^C-NMR (CDCl_3_) δ 26.5 (2C, CCH_2_CH_2_C), 29.3 (2C, CCH_2_C), 42.6 (2C, CH_2_NPh), 53.2 (2C, CH_2_N), 54.0 (2C, CH_2_N), 60.1 (2C, PhCH_2_N), 67.6 (2C, CH_2_O), 69.5 (2C, CH_2_O), 70.7 (2C, CH_2_O), 71.0 (4C, CH_2_O), 112.3 (4C, C3(Ph), C3'(Ph)), 127.5 (2C, C1(Ph)), 131.6 (4C, C2(Ph), C2'(Ph)), 148.8 (2C, C4(Ph)). HRMS (MALDI-TOF): C_34_H_55_N_4_O_5_ (M+H)^+^ calcd.; 599.4172 observed; 599.4131.

*11,16,29,32,45,50,63,66,71,80-Decaoxa-1,7,20,26,35,41,54,60-ocatazaheptacyclo-[58.8.5.5^26,35^.2^3,6^.2^21,24^.2^37,40^.1^55,58^]hexaoctaconta-3,5,21,23,37,39,55,57,74,76,83,85-dodecaene* (**20i**). Obtained as the second product in the synthesis of macrobicycle **16i**. Eluent CH_2_Cl_2_–MeOH = 5:1. Yield 15 mg (10%) of a yellow glassy compound. ^1^H-NMR (CDCl_3_) δ 1.66 (br.s, 8H, CCH_2_CH_2_C), 1.86 (quintet, *^3^J* = 5.7 Hz, 8H, NCCH_2_CN), 2.47 (br.s, 8H, CH_2_N), 3.06 (br.s, 8H, CH_2_N), 3.19 (t, *^3^J* = 5.3 Hz, 8H, CH_2_NPh), 3.37-3.45 (m, 8H, CH_2_O), 3.47-3.72 (m, 32H, CH_2_O, PhCH_2_N), 3.88 (br.s, 8H, CH_2_O), 4.45 (br.s, 4H, NH), 6.57 (d, *^3^J_obs_* = 8.0 Hz, 8H, H3(Ph), H3'(Ph)), 7.23 (d, *^3^J_obs_* = 8.0 Hz, 8H, H2(Ph), H2'(Ph)). ^13^C-NMR (CDCl_3_) δ 26.7 (4C, CCH_2_CH_2_C), 29.6 (4C, CCH_2_C), 42.3 (4C, CH_2_NPh), 51.9 (4C, CH_2_N), 54.9 (4C, CH_2_N), 60.1 (4C, PhCH_2_N), 67.2 (8C, CH_2_O), 69.1 (4C, CH_2_O), 69.4 (4C, CH_2_O), 70.0 (4C, CH_2_O), 112.5 (8C, C3(Ph), C3'(Ph)), 125.2 (4C, C1(Ph)), 131.4 (8C, C2(Ph), C2'(Ph)), 148.1 (4C, C4(Ph)). HRMS (MALDI-TOF): C_68_H_109_N_8_O_10_ (M+H)^+^ calcd.; 1197.8266 observed; 1197.8215.

*11,14,17,30,33,38-Hexaoxa-1,7,21,27-tetraazatetracyclo[25.8.5.2^3,6^.2^22,25^]tetratetraconta-3,5,22,24, 41,43-hexaene* (**16k**). Obtained from compound **7** (0.25 mmol, 139 mg), trioxadiamine **10k** (0.25 mmol, 55 mg) in the presence of Pd(dba)_2_ (23 mg, 16 mol%), BINAP (28 mg, 18 mol%), NaO*t-*Bu (0.75 mmol, 72 mg) in abs. dioxane (12 mL). Eluent CH_2_Cl_2_–MeOH = 5:1. Yield 15 mg (10%) of a yellow glassy compound. ^1^H-NMR (CDCl_3_) δ 1.85 (quintet, *^3^J* = 5.3 Hz, 4H, CCH_2_C), 2.90–3.12 (m, 8H, CH_2_N), 3.21 (t, *^3^J* = 5.5 Hz, 4H, CH_2_NPh), 3.52–3.73 (m, 24H, CH_2_O, PhCH_2_N), 3.82–3.94 (m, 4H, CH_2_O), 6.46 (d, *^3^J_obs_* = 7.9 Hz, 4H, H3(Ph), H3'(Ph)), 7.16 (d, *^3^J_obs_* = 7.9 Hz, 4H, H2(Ph), H2'(Ph)), NH protons were not assigned. ^13^C-NMR (CDCl_3_) δ 28.6 (2C, CCH_2_C), 41.7 (2C, CH_2_NPh), 52.8 (2C, CH_2_N), 54.1 (2C, CH_2_N), 59.9 (2C, PhCH_2_N), 69.7 (2C, CH_2_O), 69.9 (2C, CH_2_O), 70.2 (4C, CH_2_O), 70.6 (4C, CH_2_O), 112.3 (4C, C3(Ph), C3'(Ph)), 127.5 (2C, C1(Ph)), 131.8 (4C, C2(Ph), C2'(Ph)), 148.8 (2C, C4(Ph)). HRMS (MALDI-TOF): C_34_H_55_N_4_O_6_ (M+H)^+^ calcd.; 615.4121 observed; 615.4063.

*11,14,17,30,33,46,49,52,66,69,74,82-Dodecaoxa-1,7,21,27,36,42,56,63-octaazaheptacyclo-[61.8.5. 5^27,36^.2^3,6^.2^22,25^.2^38,41^.1^57,61^]octaoctaconta-3,5,22,24,38,40,57(77),58,60,78,85,87-dodecaene* (**20k**). Obtained as the second product in the synthesis of macrobicycle **16k**. Eluent CH_2_Cl_2_–MeOH = 5:1. Yield 29 mg (19%) of a yellow glassy compound. ^1^H-NMR (CDCl_3_) δ 1.86 (quintet, *^3^J* = 5.8 Hz, 8H, CCH_2_C), 2.45 (br.s, 8H, CH_2_N), 3.04 (br.s, 8H, CH_2_N), 3.18 (t, *^3^J* = 6.3 Hz, 8H, CH_2_NPh), 3.38–3.72 (m, 48H, CH_2_O, PhCH_2_N), 3.89 (br.s, 8H, CH_2_O), 4.49 (br.s, 4H, NH), 6.56 (d, *^3^J_obs_* = 7.8 Hz, 8H, H3(Ph), H3'(Ph)), 7.21 (d, *^3^J_obs_* = 7.8 Hz, 8H, H2(Ph), H2'(Ph)). ^13^C-NMR (CDCl_3_) δ 29.0 (4C, CCH_2_C), 42.1 (4C, CH_2_NPh), 52.0 (4C, CH_2_N), 54.9 (4C, CH_2_N), 60.2 (4C, PhCH_2_N), 67.2 (4C, CH_2_O), 67.6 (4C, CH_2_O), 69.0 (4C, CH_2_O), 70.0 (4C, CH_2_O), 70.1 (4C, CH_2_O), 70.4 (4C, CH_2_O), 112.5 (8C, C3(Ph), C3'(Ph)), 125.1 (4C, C1(Ph)), 131.4 (8C, C2(Ph), C2'(Ph)), 148.0 (4C, C4(Ph)). MS (MALDI-TOF): C_68_H_109_N_8_O_12_ (M+H)^+^ calcd.; 1229.82 observed; 1229.83.

*26,29,34,37-Tetraoxa-1,8,12,16,23-pentaazatetracyclo[21.8.8.1^3,7^.1^17,21^]hentetraconta-3(41),4,6,17 (40),18,20-hexaene* **(17d)**. Obtained from compound **8** (0.25 mmol, 150 mg), triamine **10d** (0.25 mmol, 33 mg) in the presence of Pd(dba)_2_ (12 mg, 8 mol%), BINAP (14 mg, 9 mol%), NaO*t-*Bu (0.75 mmol, 72 mg) in abs. dioxane (12 mL). Eluent CH_2_Cl_2_–MeOH–NH_3_(aq) = 100:25:5. Yield 35 mg (25%) of a yellow glassy compound. ^1^H-NMR (CDCl_3_) δ 1.78 (quintet, *^3^J* = 6.3 Hz, 4H, CCH_2_C), 2.75 (t, *^3^J* = 6.4 Hz, 4H, CH_2_NHCH_2_), 2.76 (t, *^3^J* = 5.6 Hz, 8H, CH_2_N), 3.21 (t, *^3^J* = 6.4 Hz, 4H, CH_2_NPh), 3.56–3.62 (m, 20H, CH_2_O, PhCH_2_N), 6.43 (dd, *^3^J* = 7.8 Hz, *^4^J* = 1.9 Hz, 2H, H6(Ph) or H4(Ph)), 6.51 (d, *^3^J* = 7.3 Hz, 2H, H4(Ph) or H6(Ph)), 6.88 (br.s, 2H, H2(Ph)), 7.06 (t, *^3^J* = 7.7 Hz, 2H, H5(Ph)), NH protons were not assigned. ^13^C-NMR (CDCl_3_) δ 24.2 (2C, CCH_2_C), 42.9 (2C, CH_2_NPh), 48.2 (2C, CH_2_NHCH_2_), 54.6 (4C, CH_2_N), 60.0 (2C, PhCH_2_N), 70.0 (4C, CH_2_O), 70.6 (4C, CH_2_O), 110.2 (2C, CH(Ph)), 113.7 (2C, CH(Ph)), 117.1 (2C, CH(Ph)), 128.8 (2C, C5(Ph)), 140.9 (2C, C1(Ph)), 148.7 (2C, C3(Ph)). HRMS (MALDI-TOF): C_32_H_52_N_5_O_4_ (M+H)^+^ calcd.; 570.4019 observed; 530.3976.

*26,29,57,60,65,68,75,78-Octaoxa-1,8,12,16,23,32,39,43,47,54-decaazaheptacyclo-[52.8.8.8^32,32^.1^3,7^.1^17,21^.1^34,38^.1^48,52^]dooctaconta-3(82),4,6,17(81),18,20,34(72),35,37,48(71),49,51-dodecaene* (**21d**). Obtained as the second product in the synthesis of macrobicycle **17d** Eluent CH_2_Cl_2_–MeOH–NH_3_(aq) = 100:35:6. Yield 9 mg (6%) of a yellow glassy compound. ^1^H-NMR (CDCl_3_) δ 1.79 (br.s, 8H, CCH_2_C), 2.73 (t, *^3^J* = 6.3 Hz, 8H, CH_2_NHCH_2_), 2.76–2.82 (m, 16H, CH_2_N), 3.16 (t, *^3^J* = 6.1 Hz, 8H, CH_2_NPh), 3.54–3.62 (m, 40H, CH_2_O, PhCH_2_N), 6.45 (d, *^3^J* = 7.8 Hz, 4H, H6(Ph) or H4(Ph)), 6.60 (d, *^3^J_obs_* = 6.3 Hz, 4H, H4(Ph) or H6(Ph)), 6.67 (br.s, 4H, H2(Ph)), 7.05 (t, *^3^J* = 7.6 Hz, 4H, H5(Ph)), NH protons were not assigned. ^13^C-NMR (CDCl_3_) δ 29.6 (4C, CCH_2_C), 42.7 (4C, CH_2_NPh), 48.3 (4C, CH_2_NHCH_2_), 53.8 (8C, CH_2_N), 60.2 (4C, PhCH_2_N), 70.1 (8C, CH_2_O), 70.7 (8C, CH_2_O), 111.1 (4C, CH(Ph)), 113.3 (4C, CH(Ph)), 117.8 (4C, CH(Ph)), 129.0 (4C, C5(Ph)), 140.7 (4C, C1(Ph)), 148.6 (4C, C3(Ph)). MS (MALDI-TOF): C_64_H_103_N_10_O_8_ (M+H)^+^ calcd.; 1139.80 observed; 1139.79.

*29,32,37,40-Tetraoxa-1,8,12,15,19,26-hexaazatetracyclo[24.8.8.1^3,7^.1^20,24^]tetratetraconta-3(44),4,6,20 (43),21,23-hexaene* (**17f**). Obtained from compound **8** (0.25 mmol, 150 mg), tetraamine **10f** (0.25 mmol, 44 mg) in the presence of Pd(dba)_2_ (12 mg, 8 mol%), BINAP (14 mg, 9 mol%), NaO*t-*Bu (0.75 mmol, 72 mg) in abs. dioxane (12 mL). Eluent CH_2_Cl_2_–MeOH–NH_3_(aq) = 100:25:5–100:35:6. Yield 15 mg (10%) of a yellow glassy compound. ^1^H-NMR (CDCl_3_) δ 1.77 (quintet, *^3^J* = 5.7 Hz, 4H, CCH_2_C), 2.58–2.85 (m, 16H, CH_2_N), 3.15 (t, *^3^J* = 5.9 Hz, 4H, CH_2_NPh), 3.45-3.70 (m, 20H, CH_2_O, PhCH_2_N), 6.44 (d, *^3^J* = 7.9 Hz, 2H, H6(Ph) or H4(Ph)), 6.56 (d, *^3^J* = 7.2 Hz, 2H, H4(Ph) or H6(Ph)), 6.82 (br.s, 2H, H2(Ph)), 7.05 (t, *^3^J* = 7.7 Hz, 2H, H5(Ph)), NH protons were not assigned. ^13^C-NMR (CDCl_3_) δ 28.8 (2C, CCH_2_C), 42.7 (2C, CH_2_NPh), 47.9 (2C, CH_2_NHCH_2_), 48.2 (2C, CH_2_NHCH_2_), 54.5 (4C, CH_2_N), 60.0 (2C, PhCH_2_N), 69.8 (4C, CH_2_O), 70.6 (4C, CH_2_O), 110.5 (2C, CH(Ph)), 113.7 (2C, CH(Ph)), 117.4 (2C, CH(Ph)), 128.9 (2C, C5(Ph)), 139.5 (2C, C1(Ph)), 145.8 (2C, C3(Ph)). HRMS (MALDI-TOF): C_34_H_57_N_6_O_4_ (M+H)^+^ calcd.; 613.4441 observed; 613.4412.

*29,32,63,66,71,74,81,84-Octaoxa-1,8,12,15,19,26,35,42,46,49,53,60-dodecaazaheptacyclo-[58.8.8.8^26,35^.1^3,7^.1^20,24^.1^37,41^.1^54,58^]octaoctaconta-3(88),4,6,20(87),21,23,37(78),38,40,54(77),55,57-dodecaene* (**21f**). Obtained as the second product in the synthesis of macrobicycle **17d**. Eluent CH_2_Cl_2_–MeOH–NH_3_(aq) = 100:35:6. Yield 8 mg (5%) of a yellow glassy compound. ^1^H-NMR (CDCl_3_) δ 1.76 (br.s, 8H, CCH_2_C), 2.68–2.81 (m, 32H, CH_2_N), 3.15 (br.s, 8H, CH_2_NPh), 3.50–3.63 (m, 40H, CH_2_O, PhCH_2_N), 6.43 (d, *^3^J_obs_* = 7.2 Hz, 4H, H6(Ph) or H4(Ph)), 6.60 (br.s, 4H, H2(Ph)), 6.62 (d, *^3^J* = 8.0 Hz, 4H, H4(Ph) or H6(Ph)), 7.05 (t, *^3^J* = 7.5 Hz, 4H, H5(Ph)), NH protons were not assigned. MS (MALDI-TOF): C_68_H_113_N_12_O_8_ (M+H)^+^ calcd.; 1225.88 observed; 1125.90.

*11,14,17,31,34,40-Hexaoxa-1,8,21,28-tetraazatetracyclo[26.8.6.1^3,7^.1^22,26^]tetratetraconta-3(44),4,6,22 (43),23,25-hexaene* (**17h**). Obtained from compound **8** (0.25 mmol, 150 mg), dioxadiamine **10h** (0.25 mmol, 37 mg) in the presence of Pd(dba)_2_ (12 mg, 8 mol%), BINAP (14 mg, 9 mol%), NaO*t-*Bu (0.75 mmol, 72 mg) in abs. dioxane (12 mL). Eluent CH_2_Cl_2_–MeOH = 10:1–3:1. Yield 83 mg (57%) of a yellowish glassy compound. ^1^H-NMR (CDCl_3_) δ 2.73 (br.s, 8H, CH_2_N), 3.23 (t, *^3^J* = 5.1 Hz, 4H, CH_2_NPh), 3.34 (br.s, 4H, CH_2_O), 3.51 (br.s, 8H, CH_2_O), 3.58 (t, *^3^J* = 4.9 Hz, 8H, CH_2_O), 3.62 (s, 4H, PhCH_2_N), 3.68 (t, *^3^J* = 5.1 Hz, 4H, CH_2_O), 6.45 (br.s, 4H, H(Ph)), 6.47 (d, *^3^J* = 8.0 Hz, 2H, H(Ph)), 7.07 (t, *^3^J* = 7.8 Hz, 2H, H5(Ph)), NH protons were not assigned. ^13^C-NMR (CDCl_3_) δ 43.6 (2C, CH_2_NPh), 52.8 (4C, CH_2_N), 59.6 (2C, PhCH_2_N), 67.3 (4C, CH_2_O), 68.3 (4C, CH_2_O), 69.4 (2C, CH_2_O), 70.2 (2C, CH_2_O), 110.9 (2C, CH(Ph)), 115.8 (2C, CH(Ph)), 118.0 (2C, CH(Ph)), 129.3 (2C, C5(Ph)), 138.1 (2C, C1(Ph)), 149.0 (2C, C3(Ph)). HRMS (ESI-TOF): C_32_H_51_N_4_O_6_ (M+H)^+^ calcd.; 587.3809 observed; 587.3815.

*12,17,31,34,39,42-Hexaoxa-1,8,21,28-tetraazatetracyclo[26.8.8.1^3,7^.1^22,26^]hexatetraconta-3(46),4,6,22 (45),23,25-hexaene* (**17i**). Obtained from compound **8** (0.25 mmol, 150 mg), dioxadiamine **10i** (0.25 mmol, 51 mg) in the presence of Pd(dba)_2_ (12 mg, 8 mol%), BINAP (14 mg, 9 mol%), NaO*t-*Bu (0.75 mmol, 72 mg) in abs. dioxane (12 mL). Eluent CH_2_Cl_2_–MeOH = 3:1, CH_2_Cl_2_–MeOH–NH_3_(aq) = 100:20:1. Yield 45 mg (28%) as a yellowish glassy compound. ^1^H-NMR (CDCl_3_) δ 1.65–1.71 (m, 4H, CCH_2_CH_2_C), 1.87 (quintet, *^3^J* = 6.0 Hz, 4H, CCH_2_C), 2.77 (t, *^3^J* = 5.2 Hz, 8H, CH_2_N), 3.21 (t, *^3^J* = 6.3 Hz, 4H, CH_2_NPh), 3.42–3.48 (m, 4H, CH_2_O), 3.54 (t, *^3^J* = 5.8 Hz, 4H, CH_2_O), 3.56-3.64 (m, 20H, CH_2_O, PhCH_2_N), 6.44 (dd, *^3^J* = 8.0 Hz, *^4^J* = 1.8 Hz, 2H, H6(Ph) or H4(Ph)), 6.56 (d, *^3^J_obs_* = 7.0 Hz, 2H, H4(Ph) or H6(Ph)), 6.78 (br.s, 2H, H2(Ph)), 7.06 (t, *^3^J* = 7.7 Hz, 2H, H5(Ph)), NH protons were not assigned. ^13^C-NMR (CDCl_3_) δ 26.7 (2C, CCH_2_CH_2_C), 29.3 (2C, CCH_2_C), 42.0 (2C, CH_2_NPh), 54.4 (4C, CH_2_N), 60.1 (2C, PhCH_2_N), 69.5 (2C, CH_2_O), 70.0 (4C, CH_2_O), 70.7 (2C, CH_2_O), 70.8 (4C, CH_2_O), 110.3 (2C, CH(Ph)), 113.6 (2C, CH(Ph)), 117.2 (2C, CH(Ph)), 128.8 (2C, C5(Ph)), 140.9 (2C, C1(Ph)), 148.8 (2C, C3(Ph)). HRMS (MALDI-TOF): C_36_H_59_N_4_O_6_ (M+H)^+^ calcd.; 643.4435 observed; 643.4479.

*12,15,18,32,35,40,43-Heptaoxa-1,8,22,29-tetraazatetracyclo[27.8.8.1^3,7^.1^23,27^]heptatetraconta-3(47), 4,6,23(46),24,26-hexaene* (**17k**). Obtained from compound **8** (0.25 mmol, 150 mg), trioxadiamine **10k** (0.25 mmol, 55 mg) in the presence of Pd(dba)_2_ (12 mg, 8 mol%), BINAP (14 mg, 9 mol%), NaO*t-*Bu (0.75 mmol, 72 mg) in abs. dioxane (12 mL). Eluent CH_2_Cl_2_–MeOH = 10:1. Yield 57 mg (35%) of a yellowish glassy compound. ^1^H-NMR (CDCl_3_) δ 1.82 (quintet, *^3^J* = 6.0 Hz, 4H, CCH_2_C), 2.92 (br.s, 8H, CH_2_N), 3.18 (t, *^3^J* = 6.4 Hz, 4H, CH_2_NPh), 3.54–3.60 (m, 12H, CH_2_O), PhCH_2_N), 3.62–3.67 (m, 12H, CH_2_O), 3.76 (br.s, 4H, CH_2_O), 4.18 (br.s, 2H, NH), 6.48 (d, *^3^J_obs_* = 7.2 Hz, 2H, H4(Ph) or H6(Ph)), 6.61 (d, *^3^J_obs_* = 7.2 Hz, 2H, H6(Ph) or H4(Ph)), 6.75 (br.s, 2H, H2(Ph)), 7.05 (t, *^3^J* = 7.7 Hz, 2H, H5(Ph)). ^13^C-NMR (CDCl_3_) δ 29.0 (2C, CCH_2_C), 41.4 (2C, CH_2_NPh), 52.9 (2C, CH_2_N), 54.0 (2C, CH_2_N), 59.8 (2C, PhCH_2_N), 67.3 (4C, CH_2_O), 68.5 (4C, CH_2_O), 69.5 (2C, CH_2_O), 70.1 (2C, CH_2_O), 70.5 (2C, CH_2_O), 110.8 (2C, CH(Ph)), 115.1 (2C, CH(Ph)), 117.4 (2C, CH(Ph)), 129.3 (2C, C5(Ph)), 138.2 (2C, C1(Ph)), 149.0 (2C, C3(Ph)). HRMS (ESI-TOF): C_36_H_59_N_4_O_7_ (M+H)^+^ calcd.; 659.4384 observed; 659.4389.

*12,15,18,32,35,49,52,55,69,72,77,80,87,90-Tetradecaoxa-1,8,22,29,38,45,59,66-octaazaheptacyclo-[64.8.8.8^29,38^.1^3,7^.1^23,27^.1^40,44^.1^60,64^]tetranonaconta-3(94),4,6,23(93),24,26,40(84),41,43,60(83),61,63-dodecaene* (**21k**). Obtained as the second product in the synthesis of macrobicycle **17k**. Eluent CH_2_Cl_2_–MeOH = 10:3. Yield 28 mg (17%) of a yellow glassy compound. ^1^H-NMR (CDCl_3_) δ 1.83 (quintet, *^3^J* = 5.7 Hz, 8H, CCH_2_C), 2.81 (br.s, 16H, CH_2_N), 3.18 (t, *^3^J* = 6.3 Hz, 8H, CH_2_NPh), 3.50–3.68 (m, 64H, CH_2_O, PhCH_2_N), 4.56 (br.s, 4H, NH), 6.46 (d, *^3^J* = 7.8 Hz, 4H, H4(Ph) or H6(Ph)), 6.55 (d, *^3^J_obs_* = 7.0 Hz, 4H, H6(Ph) or H4(Ph)), 6.73 (s, 4H, H2(Ph)), 7.05 (t, *^3^J_obs_* = 7.5 Hz, 4H, H5(Ph)). MS (MALDI-TOF): C_72_H_117_N_8_O_14_ (M+H)^+^ calcd.; 1317.87 observed; 1317.84.

*10,13,25,28,33,36-Hexaoxa-1,7,16,22-tetraazatetracyclo[20.8.8.2^3,6^.2^17,20^]dotetraconta-3,5,17,19,39,41-hexaene* (**18h**). Obtained from compound **9** (0.25 mmol, 150 mg), dioxadiamine **10h** (0.25 mmol, 37 mg) in the presence of Pd(dba)_2_ (24 mg, 16 mol%), BINAP (28 mg, 18 mol%), NaO*t-*Bu (0.75 mmol, 72 mg) in abs. dioxane (12 mL). Eluent CH_2_Cl_2_–MeOH = 3:1, CH_2_Cl_2_–MeOH–NH_3_(aq) = 100:20:1–100:20:3. Yield 37 mg (25%) of a yellowish glassy compound. ^1^H-NMR (CDCl_3_) δ 2.68 (t, *^3^J* = 5.4 Hz, 8H, CH_2_N), 3.27 (t, *^3^J* = 4.8 Hz, 4H, CH_2_NPh), 3.53 (s, 4H, CH_2_O), 3.59 (t, *^3^J* = 5.4 Hz, 8H, CH_2_O), 3.62 (s, 8H, CH_2_O), 3.65 (s, 4H, PhCH_2_N), 3.73 (t, *^3^J* = 4.8 Hz, 4H, CH_2_O), 6.55 (d, *^3^J_obs_* = 8.5 Hz, 4H, H3(Ph), H3'(Ph)), 7.17 (d, *^3^J_obs_* = 8.5 Hz, 4H, H2(Ph), H2'(Ph)), NH protons were not assigned. ^13^C-NMR (CDCl_3_) δ 43.7 (2C, CH_2_NPh), 54.9 (4C, CH_2_N), 59.7 (2C, PhCH_2_N), 69.4 (2C, CH_2_O), 69.8 (4C, CH_2_O), 70.0 (2C, CH_2_O), 70.8 (4C, CH_2_O), 113.1 (4C, C3(Ph), C3'(Ph)), 128.8 (2C, C1(Ph)), 129.8 (4C, C2(Ph), C2'(Ph)), 147.2 (2C, C4(Ph)). HRMS (ESI-TOF): C_32_H_51_N_4_O_6_ (M+H)^+^ calcd.; 587.3809 observed; 587.3829.

*10,13,25,28,40,43,55,58,63,66,75,78-dodecaoxa-1,7,16,22,31,37,46,52-octaazaheptacyclo-[50.8.8.8^22,31^.2^3,6^.2^17,20^.2^33,36^.2^47,50^]tetraoctaconta-3,5,17,19,33,35,47,49,69,71,81,83-dodecaene* (**22h**). Obtained as the second product in the synthesis of macrobicycle **18h**. Eluent CH_2_Cl_2_–MeOH–NH_3_(aq) = 100:25:5. Yield 15 mg (10%) of a yellow glassy compound. ^1^H-NMR (CDCl_3_) δ 2.77 (br.s, 16H, CH_2_N), 3.26 (br.s, 8H, CH_2_NPh), 3.58 (br.s, 40H, CH_2_O), 3.63 (s, 8H, PhCH_2_N), 3.68 (br.s, 8H, CH_2_O), 3.95 (br.s, 4H, NH), 6.54 (d, *^3^J_obs_* = 8.2 Hz, 8H, H3(Ph), H3'(Ph)), 7.09 (d, *^3^J_obs_* = 8.2 Hz, 8H, H2(Ph), H2'(Ph)). HRMS (MALDI-TOF): C_64_H_101_N_8_O_12_ (M+H)^+^ calcd.; 1173.7538 observed; 1173.7472.

*11,14,17,30,33,38,41-Heptaoxa-1,7,21,27-tetraazatetracyclo[25.8.8.2^3,6^.2^22,25^]heptatetraconta-3,5,22, 24,44,46-hexaene* (**18k**). Obtained from compound **9** (0.25 mmol, 150 mg), trioxadiamine **10k** (0.25 mmol, 55 mg) in the presence of Pd(dba)_2_ (24 mg, 16 mol%), BINAP (28 mg, 18 mol%), NaO*t-*Bu (0.75 mmol, 72 mg) in abs. dioxane (12 mL). Eluent CH_2_Cl_2_–MeOH = 10:1, 3:1. Yield 59 mg (36%) as a yellowish glassy compound. ^1^H-NMR (CDCl_3_) δ 1.80 (br.s, 4H, CCH_2_C), 2.70 (br.s, 8H, CH_2_N), 3.13 (br.s, 4H, CH_2_NPh), 3.34 (br.s, 4H, CH_2_O), 3.50 (br.s, 8H, CH_2_O), 3.58 (s, 8H, CH_2_O), 3.63–3.69 (m, 12H, CH_2_O, PhCH_2_N), 6.50 (d, *^3^J_obs_* = 8.1 Hz, 4H, H3(Ph), H3'(Ph)), 6.79 (d, *^3^J_obs_* = 8.1 Hz, 4H, H2(Ph), H2'(Ph)), NH protons were not assigned. ^13^C-NMR (CDCl_3_) δ 28.9 (2C, CCH_2_C), 41.8 (2C, CH_2_NPh), 52.2 (4C, CH_2_N), 58.6 (2C, PhCH_2_N), 66.8 (4C, CH_2_O), 68.5 (4C, CH_2_O), 69.7 (2C, CH_2_O), 70.2 (2C, CH_2_O), 70.4 (2C, CH_2_O), 112.7 (4C, C3(Ph), C3'(Ph)), 124.5 (2C, C1(Ph)), 130.5 (4C, C2(Ph), C2'(Ph)), 148.4 (2C, C4(Ph)). HRMS (ESI-TOF): C_36_H_59_N_4_O_7_ (M+H)^+^ calcd.; 659.4384 observed; 659.4375.

*10,13,25,28,33-Pentaoxa-1,5,7,16,18,22-hexaazatetracyclo[20.8.5.2^3,6^.2^17,20^]nonatriaconta-3,5,17,19,36,38-hexaene* (**27h**). Obtained from compound **23** (0.5 mmol, 235 mg), dioxadiamine **10h** (0.5 mmol, 74 mg) in the presence of Pd(dba)_2_ (46 mg, 16 mol%), DavePhos (36 mg, 18 mol%), NaO*t-*Bu (1.5 mmol, 144 mg) in abs. Dioxane (25 mL). Eluent CH_2_Cl_2_–MeOH–NH_3_(aq) = 100:20:2. Yield 60 mg (22%) of a yellow glassy compound. ^1^H-NMR (CDCl_3_) δ 2.56 (t, *^3^J* = 5.1 Hz, 4H, CH_2_N), 2.66 (t, *^3^J* = 4.6 Hz, 4H, CH_2_N), 3.42 (br.s, 4H, CH_2_NPy), 3.52 (t, *^3^J* = 5.1 Hz, 4H, CH_2_O), 3.55–3.62 (m, 8H, CH_2_O), 3.66 (s, 4H, CH_2_O or PyCH_2_N), 3.67 (s, 4H, PyCH_2_N or CH_2_O), 3.72 (t, *^3^J* = 5.1 Hz, 4H, CH_2_O), 5.11 (br.s, NH), 6.30 (d, *^3^J* = 8.5 Hz, 2H, H5(Py)), 7.47 (d, *^3^J* = 8.5 Hz, 2H, H6(Py)), 7.94 (s, 2H, H2(Py)). ^13^C-NMR (CDCl_3_) δ 41.6 (2C, CH_2_NPy), 54.7 (2C, CH_2_N), 55.2 (2C, CH_2_N), 57.4 (2C, PyCH_2_N), 69.6 (4C, CH_2_O), 69.7 (2C, CH_2_O), 70.0 (2C, CH_2_O), 70.8 (2C, CH_2_O), 108.3 (2C, C5(Py)), 123.9 (2C, C1(Py)), 138.6 (2C, C6(Py)), 147.4 (2C, C2(Py)), 158.0 (2C, C4(Py)). HRMS (MALDI-TOF): C_28_H_45_N_6_O_5_ (M+H)^+^ calcd.; 545.3451 observed; 545.3480.

*10,15,27,30,35-Pentaoxa-1,5,7,18,20,24-hexaazatetracyclo[22.8.5.2^3,6^.2^19,22^]hentetraconta-3,5,19,21, 38,40-hexaene* (**27i**). Obtained from compound **23** (0.25 mmol, 117 mg), dioxadiamine **10i** (0.25 mmol, 51 mg) in the presence of Pd(dba)_2_ (23 mg, 16 mol%), DavePhos (18 mg, 18 mol%), NaO*t-*Bu (0.75 mmol, 72 mg) in abs. dioxane (12 mL). Eluent CH_2_Cl_2_–MeOH–NH_3_(aq) = 100:20:1–100:20:3. Yield 17 mg (11%) of a yellow glassy compound. ^1^H-NMR (CDCl_3_) δ 1.63–1.70 (m, 4H, CCH_2_CH_2_C), 1.87 (quintet, *^3^J* = 5.5 Hz, 4H, CCH_2_C), 2.71 (br.s, 8H, CH_2_N), 3.39 (t, *^3^J* = 5.2 Hz, 4H, CH_2_NPy), 3.42–3.48 (m, 4H, CH_2_O), 3.50–3.64 (m, 20H, CH_2_O, PyCH_2_N), 5.26 (br.s, 2H, NH), 6.35 (d, *^3^J* = 8.5 Hz, 2H, H5(Py)), 7.58 (d, *^3^J* = 8.5 Hz, 2H, H6(Py)), 7.91 (br.s, 2H, H2(Py)). ^13^C-NMR (CDCl_3_) δ 26.8 (2C, CCH_2_CH_2_C), 29.3 (2C, CCH_2_C), 40.7 (2C, CH_2_NPy), 54.4 (2C, CH_2_N), 54.5 (2C, CH_2_N), 57.4 (2C, PyCH_2_N), 69.0 (2C, CH_2_O), 69.5 (4C, CH_2_O), 70.6 (2C, CH_2_O), 71.0 (2C, CH_2_O), 107.1 (2C, C5(Py)), 122.5 (2C, C1(Py)), 139.4 (2C, C6(Py)), 147.4 (2C, C2(Py)), 158.1 (2C, C4(Py)). HRMS (MALDI-TOF): C_32_H_53_N_6_O_5_ (M+H)^+^ calcd.; 601.4077 observed; 601.4043.

*11,14,17,30,33,38-Hexaoxa-1,5,7,21,23,27-hexaazatetracyclo[25.8.5.2^3,6^.2^22,25^]tetratetraconta-3,5,22, 24,41,43-hexaene* (**27k**). Obtained from compound **23** (0.25 mmol, 117 mg), trioxadiamine **10k** (0.25 mmol, 55 mg) in the presence of Pd(dba)_2_ (23 mg, 16 mol%), DavePhos (18 mg, 18 mol%), NaO*t-*Bu (0.75 mmol, 72 mg) in abs. dioxane (12 mL). Eluent CH_2_Cl_2_–MeOH = 5:1, CH_2_Cl_2_–MeOH–NH_3_(aq) = 100:20:1–100:20:3. Yield 14 mg (9%) of a yellow glassy compound. ^1^H-NMR (CDCl_3_) δ 1.87 (quintet, *^3^J* = 5.9 Hz, 4H, CCH_2_C), 2.71 (t, *^3^J* = 5.3 Hz, 4H, CH_2_N), 2.77 (t, *^3^J* = 4.7 Hz, 4H, CH_2_N), 3.39 (br.s, 4H, CH_2_NPy), 3.51-3.70 (m, 28H, CH_2_O, PyCH_2_N), 5.64 (br.s, 2H, NH), 6.32 (d, *^3^J* = 8.6 Hz, 2H, H5(Py)), 7.54 (d, *^3^J* = 8.6 Hz, 2H, H6(Py)), 7.90 (br.s, 2H, H2(Py)). ^13^C-NMR (CDCl_3_) δ 29.0 (2C, CCH_2_C), 40.0 (2C, CH_2_NPy), 54.1 (2C, CH_2_N), 54.3 (2C, CH_2_N), 57.2 (2C, PyCH_2_N), 68.7 (2C, CH_2_O), 69.2 (2C, CH_2_O), 69.4 (2C, CH_2_O), 70.1 (2C, CH_2_O), 70.5 (2C, CH_2_O), 70.6 (2C, CH_2_O), 107.5 (2C, C5(Py)), 123.0 (2C, C1(Py)), 139.9 (2C, C6(Py)), 146.6 (2C, C2(Py)), 157.7 (2C, C4(Py)). HRMS (MALDI-TOF): C_32_H_53_N_6_O_6_ (M+H)^+^ calcd.; 617.4027 observed; 617.3967.

*10,13,25,28,33,36-Hexaoxa-1,5,7,16,18,22-hexaazatetracyclo[20.8.8.2^3,6^.2^17,20^]dotetraconta-3,5,17,19,39,41-hexaene* (**28h**). Obtained from compound **24** (0.25 mmol, 128 mg), dioxadiamine **10h** (0.25 mmol, 37 mg) in the presence of Pd(dba)_2_ (23 mg, 16 mol%), DavePhos (18 mg, 18 mol%), NaO*t-*Bu (0.75 mmol, 72 mg) in abs. dioxane (12 mL). Eluent CH_2_Cl_2_–MeOH–NH_3_(aq) = 100:20:3. Yield 7 mg (5%) as a yellow glassy compound. ^1^H-NMR (CDCl_3_) δ 2.70 (br.s, 8H, CH_2_N), 3.47–3.54 (m, 8H, CH_2_NPy, CH_2_O), 3.55–3.61 (m, 16H, CH_2_O), 3.67 (s, 4H, PyCH_2_N), 3.73 (t, *^3^J* = 4.7 Hz, 4H, CH_2_O), 5.61 (br.s, 2H, NH), 6.37 (d, *^3^J* = 8.2 Hz, 2H, H6(Py)), 7.52 (d, *^3^J* = 8.2 Hz, 2H, H5(Py)), 7.97 (br.s, 2H, H2(Py)). HRMS (ESI-TOF): C_30_H_49_N_6_O_6_ (M+H)^+^ calcd.; 589.3713 observed; 589.3715.

*11,14,17,30,33,38,41-Heptaoxa-1,5,7,21,23,27-hexaazatetracyclo[25.8.8.2^3,6^.2^22,25^]heptatetraconta-3,5,22,24,44,46-hexaene* (**28k**). Obtained from compound **24** (0.25 mmol, 128 mg), trioxadiamine **10k** (0.25 mmol, 55 mg) in the presence of Pd(dba)_2_ (23 mg, 16 mol%), DavePhos (18 mg, 18 mol%), NaO*t-*Bu (0.75 mmol, 72 mg) in abs. dioxane (12 mL). Eluent CH_2_Cl_2_–MeOH–NH_3_(aq) = 100:20:1–100:20:2. Yield 40 mg (24%) of a yellow glassy compound. ^1^H-NMR (CDCl_3_) δ 1.87 (quintet, *^3^J* = 6.0 Hz, 4H, CCH_2_C), 2.69 (t, *^3^J* = 5.5 Hz, 4H, CH_2_N), 2.79 (t, *^3^J* = 4.6 Hz, 4H, CH_2_N), 3.38 (q, *^3^J* = 6.1 Hz, 4H, CH_2_NPy), 3.52-3.69 (m, 32H, CH_2_O, PyCH_2_N), 5.20 (br.s, 2H, NH), 6.32 (d, *^3^J* = 8.6 Hz, 2H, H5(Py)), 7.47 (dd, *^3^J* = 8.6 Hz, *^4^J* = 2.0 Hz, 2H, H6(Py)), 7.89 (d, *^4^J* = 2.0 Hz, 2H, H2(Py)). ^13^C-NMR (CDCl_3_) δ 29.1 (2C, CCH_2_C), 39.8 (2C, CH_2_NPy), 54.1 (4C, CH_2_N), 56.8 (2C, PyCH_2_N), 69.4 (2C, CH_2_O), 69.9 (4C, CH_2_O), 70.2 (4C, CH_2_O), 70.5 (2C, CH_2_O), 70.8 (2C, CH_2_O), 107.1 (2C, C5(Py)), 123.1 (2C, C1(Py)), 138.8 (2C, C6(Py)), 147.7 (2C, C2(Py)), 158.2 (2C, C4(Py)). HRMS (ESI-TOF): C_34_H_57_N_6_O_7_ (M+H)^+^ calcd.; 661.4288 observed; 661.4241.

*12,15,18,32,35,40-Hexaoxa-1,8,22,29,43,44-hexaazatetracyclo[27.8.5.1^3,7^.1^23,27^]tetratetraconta-3(44), 4,6,23(43),24,26-hexaene* (**29k**). Obtained from compound **25** (0.25 mmol, 140 mg), trioxadiamine **10k** (0.25 mmol, 55 mg) in the presence of Pd(dba)_2_ (23 mg, 16 mol%), BINAP (28 mg, 18 mol%), NaO*t-*Bu (0.75 mmol, 72 mg) in abs. dioxane (12 mL). Eluent CH_2_Cl_2_–MeOH = 3:1. Yield 21 mg (12%) of a yellow glassy compound. ^1^H-NMR (CDCl_3_) δ 2.02 (br.s, 4H, CCH_2_C), 2.60–2.88 (m, 8H, CH_2_N), 3.15 (q, *^3^J* = 5.6 Hz, 4H, CH_2_NPy), 3.28 (t, *^3^J* = 5.6 Hz, 4H, CH_2_O), 3.31–3.38 (m, 4H, CH_2_O), 3.40–3.65 (m, 20H, CH_2_O, PyCH_2_N), 5.82 (t, *^3^J* = 4.8 Hz, 2H, NH), 6.41 (d, *^3^J* = 8.5 Hz, 2H, H4(Py) or H6(Py)), 6.51 (d, *^3^J* = 7.1 Hz, 2H, H6(Py) or H4(Py)), 7.45 (dd, *^3^J* = 8.5 Hz, *^3^J* = 7.1 Hz, 2H, H5(Py)). ^13^C-NMR (CDCl_3_) δ 28.8 (2C, CCH_2_C), 39.3 (2C, CH_2_NPy), 53.8 (2C, CH_2_N), 56.1 (2C, CH_2_N), 61.9 (2C, PyCH_2_N), 66.8 (2C, CH_2_O), 66.9 (2C, CH_2_O), 67.5 (2C, CH_2_O), 69.0 (2C, CH_2_O), 70.3 (2C, CH_2_O), 70.6 (2C, CH_2_O), 105.1 (2C, C4(Py) or C6(Py)), 112.5 (2C, C6(Py) or C4(Py)), 139.3 (2C, C5(Py)), 156.4 (2C, C1(Py)), 160.1 (2C, C3(Py)). HRMS (MALDI-TOF): C_32_H_52_N_6_NaO_6_ (M+Na)^+^ calcd.; 639.3846 observed; 639.3803.

*11,14,27,30,35,38-Hexaoxa-1,8,17,24,41,42-hexaazatetracyclo[22.8.8.1^3,7^.1^18,22^]dotetraconta-3(42),4, 6,18(41),19,21-hexaene* (**30h**). Obtained from compound **26** (0.227 mmol, 137 mg), dioxadiamine **10h** (0.23 mmol, 34 mg) in the presence of Pd(dba)_2_ (21 mg, 16 mol%), DavePhos (16 mg, 18 mol%), NaO*t-*Bu (0.75 mmol, 72 mg) in abs. dioxane (12 mL). Eluent CH_2_Cl_2_–MeOH = 3:1. Yield 21 mg (16%) of a yellow glassy compound. ^1^H-NMR (CDCl_3_) δ 2.64 (br.s, 8H,CH_2_N), 3.03 (br.s, 4H, CH_2_NPy), 3.30 (s, 4H, CH_2_O), 3.33–3.38 (m, 8H, CH_2_O), 3.47 (t, *^3^J* = 4.5 Hz, 4H, CH_2_O), 3.50–3.68 (m, 12H, CH_2_O, PyCH_2_N), 6.42 (d, *^3^J* = 8.5 Hz, 2H, H4(Py) or H6(Py)), 6.51 (d, *^3^J* = 7.2 Hz, 2H, H6(Py) or H4(Py)), 7.46 (t, *^3^J* = 7.8 Hz, 2H, H5(Py)), NH protons were not assigned. ^13^C-NMR (CDCl_3_) δ 42.5 (2C, CH_2_NPy), 52.3 (4C, CH_2_N), 60.4 (2C, PyCH_2_N), 67.1 (4C, CH_2_O), 67.3 (4C, CH_2_O), 68.4 (2C, CH_2_O), 69.8 (2C, CH_2_O), 105.4 (2C, C4(Py) or C6(Py)), 112.8 (2C, C6(Py) or C4(Py)), 138.8 (2C, C5(Py)), 158.2 (2C, C1(Py)), 161.7 (2C, C3(Py)). HRMS (MALDI-TOF): C_30_H_49_N_6_O_6_ (M+H)^+^ calcd.; 589.3714 observed; 589.3675.

## 4. Conclusions

We can conclude that as a result of this investigation, we have elaborated a convenient and versatile approach to a previously unknown family of macrobicycles based on diazacrown ethers possessing additional diamine, oxadiamine and tetraamine chains using Pd-catalyzed amination reactions. We established the dependence of the yields of target cryptands on the nature of the starting compounds and found out that in the case of macrobicycles possessing benzyl spacers, quite good yields of the products as high as 57% can be achieved, mainly with *meta*-aminobenzyl spacers, especially with triamine and oxadiamine linkers, while in the case of macrobicycles with pyridyl spacers, the yields are generally substantially lower and hardly surpass 20%. A number of compounds synthesized in this work are now under investigation concerning their coordination properties towards various metal cations.
